# A Novel Core Genome-Encoded Superantigen Contributes to Lethality of Community-Associated MRSA Necrotizing Pneumonia

**DOI:** 10.1371/journal.ppat.1002271

**Published:** 2011-10-13

**Authors:** Gillian J. Wilson, Keun Seok Seo, Robyn A. Cartwright, Timothy Connelley, Olivia N. Chuang-Smith, Joseph A. Merriman, Caitriona M. Guinane, Joo Youn Park, Gregory A. Bohach, Patrick M. Schlievert, W. Ivan Morrison, J. Ross Fitzgerald

**Affiliations:** 1 The Roslin Institute and Centre for Infectious Diseases, University of Edinburgh, Easter Bush Campus, Midlothian, United Kingdom; 2 Department of Basic Sciences, Mississippi State University, Mississippi State, Mississippi, United States of America; 3 Department of Microbiology, University of Minnesota Medical School, Minneapolis, Minnesota, United States of America; 4 Department of Biochemistry and Molecular Biology, Mississippi State University, Mississippi State, Mississippi, United States of America; National Institute of Allergy and Infectious Diseases, National Institutes of Health, United States of America

## Abstract

Bacterial superantigens (SAg) stimulate T-cell hyper-activation resulting in immune modulation and severe systemic illnesses such as *Staphylococcus aureus* toxic shock syndrome. However, all known *S. aureus* SAgs are encoded by mobile genetic elements and are made by only a proportion of strains. Here, we report the discovery of a novel SAg staphylococcal enterotoxin-like toxin X (SElX) encoded in the core genome of 95% of phylogenetically diverse *S. aureus* strains from human and animal infections, including the epidemic community-associated methicillin-resistant *S. aureus* (CA-MRSA) USA300 clone. SElX has a unique predicted structure characterized by a truncated SAg B-domain, but exhibits the characteristic biological activities of a SAg including Vβ-specific T-cell mitogenicity, pyrogenicity and endotoxin enhancement. In addition, SElX is expressed by clinical isolates *in vitro,* and during human, bovine, and ovine infections, consistent with a broad role in *S. aureus* infections of multiple host species. Phylogenetic analysis suggests that the *selx* gene was acquired horizontally by a progenitor of the *S. aureus* species, followed by allelic diversification by point mutation and assortative recombination resulting in at least 17 different alleles among the major pathogenic clones. Of note, SElX variants made by human- or ruminant-specific *S. aureus* clones demonstrated overlapping but distinct Vβ activation profiles for human and bovine lymphocytes, indicating functional diversification of SElX in different host species. Importantly, SElX made by CA-MRSA USA300 contributed to lethality in a rabbit model of necrotizing pneumonia revealing a novel virulence determinant of CA-MRSA disease pathogenesis. Taken together, we report the discovery and characterization of a unique core genome-encoded superantigen, providing new insights into the evolution of pathogenic *S. aureus* and the molecular basis for severe infections caused by the CA-MRSA USA300 epidemic clone.

## Author Summary

*Staphylococcus aureus* is a global pathogen, responsible for an array of different illnesses in humans and animals. In particular, community-associated methicillin-resistant *S. aureus* (CA-MRSA) strains of the pandemic USA300 clone have the capacity to cause lethal human necrotizing pneumonia, but the molecular basis for the enhanced virulence remains unclear. Bacterial superantigens (SAg) stimulate T-cell hyper-activation resulting in severe systemic illnesses such as toxic shock syndrome (TSS). However, all *S. aureus* SAgs identified to date are encoded by mobile genetic elements found only in a proportion of clinical isolates. Here, we report the discovery of a unique core genome-encoded SAg (SElX) which was acquired by an ancestor of the *S. aureus* species and which has undergone genetic and functional diversification in pathogenic clones infecting humans and animals. Importantly, we report that SElX made by pandemic USA300 contributes to lethality in a rabbit model of human necrotizing pneumonia revealing a novel virulence determinant of severe CA-MRSA infection.

## Introduction

*Staphylococcus aureus* is responsible for an array of diseases including life-threatening toxinoses such as toxic shock syndrome (TSS) and necrotizing pneumonia. Many strains of *S. aureus* have been shown to produce members of a family of more than 20 serologically distinct superantigenic (SAg) toxins, which contribute to disease pathogenesis via modulation of the host immune response [1], [2]. Previously characterized SAgs are small secreted proteins of 20 to 28 kDa in size, which share similar biochemical, structural, and immunobiological properties [1], [2], but can be differentiated into 4 distinct subgroups according to their phylogenetic relatedness [3]–[5]. They share a compact 2-domain protein structure consisting of domain A which contains a long central α-helix and a C-terminal β-grasp motif, and the smaller domain B which contains an N-terminal oligonucleotide-oligosaccharide binding (OB) fold [1], [2], [5], [6]. SAgs bypass the conventional antigen processing pathway, by binding simultaneously to MHC class II molecules of antigen-presenting cells, outside of the antigen-binding groove, and the variable region of the T-cell receptor (TCR) β-chain (Vβ) [7], [8]. In so doing, SAgs can react with all T-cells expressing reactive Vβ-TCR regions (up to 50%), instead of the normal responding frequency to an antigen of approximately 0.01%, resulting in massive inflammatory cytokine release and consequent toxic shock [9]. SAgs also have the capacity to cause immune suppression by inducing T-cell anergy, and may contribute to bacterial persistence during chronic infection [10].

All staphylococcal SAgs identified to date are encoded by mobile genetic elements (MGE) such as plasmids, phages, transposons and *S. aureus* pathogenicity islands (SaPIs), or the highly variable genomic region *v*Saβ [4], [11]–[14]. Accordingly, the distribution of SAg genes is *S. aureus* strain-dependent. For example, Omoe *et al.* reported that 80% of human isolates contain at least one SAg gene, including 50% which contain the enterotoxin gene cluster (*egc)* locus, and Smyth *et al.* demonstrated that 57% of animal strains examined contained at least one SAg gene with the *egc* found in 30% of isolates [15], [16]. These data collectively suggest that no single SAg is encoded by more than 50% of strains and that some strains may not have superantigenic capacity at all. The USA300 *S. aureus* clone which is responsible for the current CA-MRSA epidemic affecting the USA and other countries is typically associated with skin and soft tissue infections and has the capacity to cause lethal toxinoses such as necrotizing pneumonia and extreme pyrexia [17]–[19]. However, USA300 strains do not typically produce TSST-1 or staphylococcal enterotoxins B and C, the SAgs most often associated with toxic shock [20], [21]. Here, we report the discovery of a unique core genome-encoded SAg (designated SElX), encoded by the great majority of *S. aureus* strains, determine its immunobiological function and examine its molecular evolution. Our data suggest that SElX contributes to immune modulation in both human and animal disease pathogenesis, and demonstrate a role for SElX in the development CA-MRSA USA300 necrotizing pneumonia in a rabbit model of infection.

## Results

### Identification of a novel putative SAg gene encoded by the great majority of *S. aureus* strains

**Figure 1:**
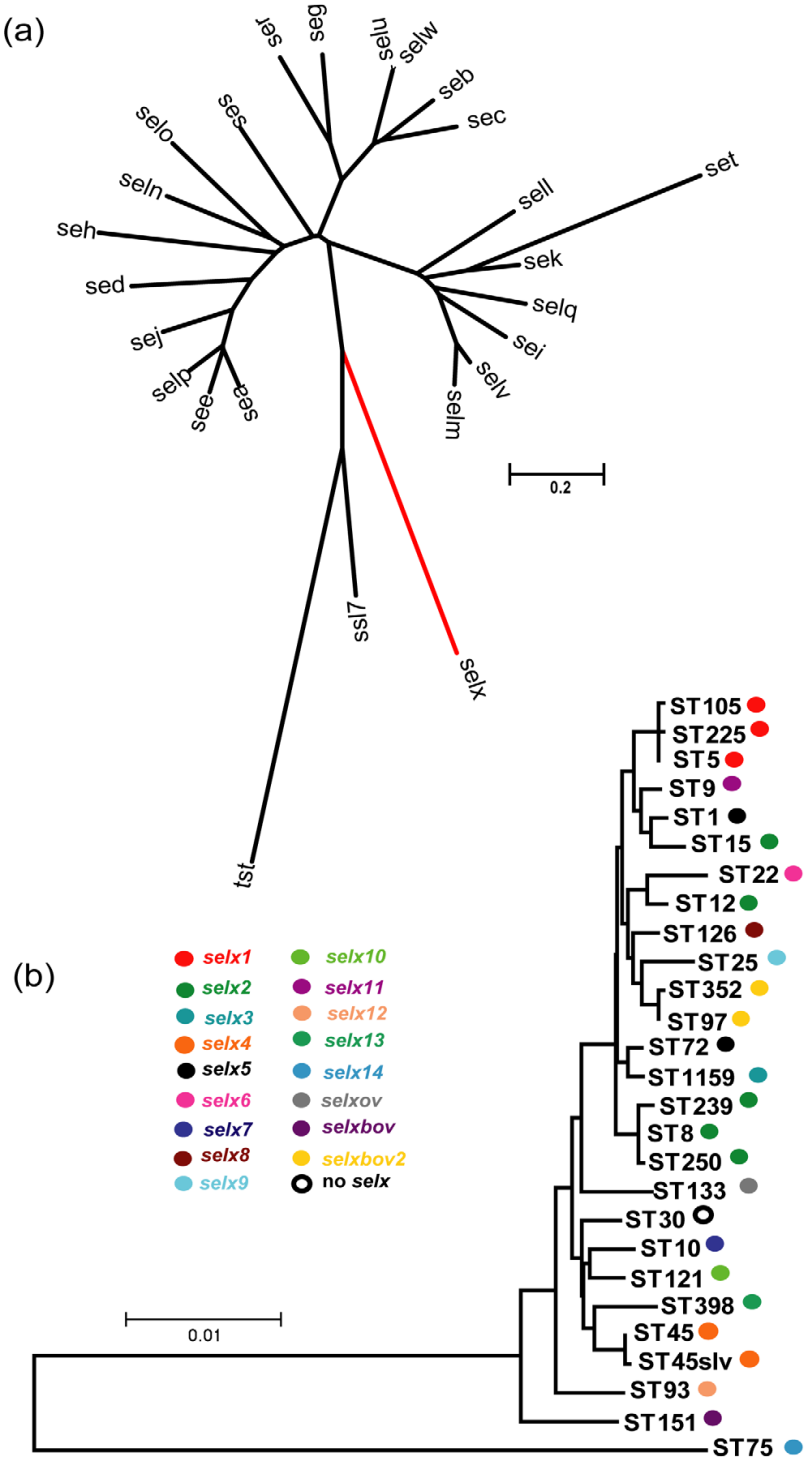
Phylogenetic analysis of *selx* and its species-wide distribution. **a**) Neighbor*-*joining tree based on the sequences of all known staphylococcal SAgs and the SAg-like protein, SSL7 **b**) Neighbor-joining tree of concatenated MLST sequences of representative *S. aureus* STs representing the breadth of species diversity. Coloured circles denote the presence of specific *selx* alleles.

In order to examine the superantigenic capacity of the epidemic CA-MRSA clone, USA300, we carried out a genome-wide screen of USA300 strain FPR3757 to identify genes encoding proteins with homology to known SAgs [21]. In addition to characterized SAgs, SElK and SElQ, we discovered a SAg gene homolog, SAUSA300_0370 which encodes a protein with 24% amino acid identity to the major virulence factor, TSST-1 and 27% identity with the SAg-like protein, SSL7. The gene, designated *selx* encoding staphylococcal enterotoxin-like toxin X (SElX), is 612 bp in length and is most closely-related to the phylogenetic group IV of staphylococcal SAgs represented by TSST-1, closely allied to the staphylococcal SAg-like (SSL) protein family (Fig. 1a). The mature form of SElX is 168 amino acids in length with a predicted molecular weight of 19343 Da, smaller than any known staphylococcal SAg [1], [4]. In order to examine the distribution of *selx* among *S. aureus* strains, we screened a total of 114 isolates by a combination of bioinformatic interrogation of 53 publicly available *S. aureus* genome sequences (Table S1), and PCR analysis using *selx*-specific oligonucleotide primers (Table S2) of a further 61 *S. aureus* isolates selected to represent the full breadth of species diversity and to include the most important human and animal pathogenic clones (Table S3). Remarkably, 108 of the 114 isolates (95%) contained the *selx* gene which included all strains examined except isolates of the CC30 clonal complex (Fig. 1b; Table S1 and S3).

Analysis of the location of *selx* in the genomes of strains representing diverse multi-locus sequence types (ST)s, including MSSA476 (ST1), N315 (ST5), USA300 TCH3757 (ST8), H19 (ST10), A9635 (ST45slv), TCH130 (ST72), JKD6159 (ST93), JH1 (ST105), ED133 (ST133), RF122 (ST151), 04–02891 (ST225), JKD6008 (ST239), COL (ST250) and TCH959 (ST1159), revealed that *selx* is located at an identical chromosomal site in all strains examined (Fig. S1). Specifically, it is located ∼400 kb from the origin of replication in the *oriC* environ among a cluster of 4 genes specific for the *S. aureus* species including 2 genes encoding hypothetical proteins of unknown function, and a predicted integrase pseudogene which contains partial homology to an integrase encoded by the *S. aureus* bacteriophage, PT1028 [22]. The gene cluster is flanked by conserved genes encoding ribosomal proteins and a DNA-binding protein involved in DNA replication, which are located at the same genomic location in other staphylococcal species such as *Staphylococcus epidermidis* (Fig. S1). The genetic linkage of *selx* with an integrase pseudogene, its wide distribution across the full breadth of *S. aureus* species diversity, and its absence in the genomes of other staphylococcal species indicates an ancient horizontal acquisition which may have occurred during *S. aureus* speciation. The existence of a single *S. aureus* clonal complex (CC30), which does not contain *selx* but contains the adjacent integrase pseudogene at the same chromosomal site, is consistent with a deletion event in a progenitor of the clonal lineage which contained the *selx* gene (Fig. S1).

### *selx* exhibits considerable species-wide allelic variation and evidence for assortative recombination

In order to examine the allelic variation of *selx* among *S. aureus* strains representing the breadth of species diversity, DNA sequencing of *selx* was carried out for 11 *S. aureus* strains representative of ST 9, 12, 15, 25, 45, 97, 121, and 126 and compared to the sequence of *selx* for 49 strains available in Genbank (Table S1). *selx* was represented by 17 distinct allelic variants encoded by human and animal strains, designated *selx1-14*, *selxbov1-2, and selxov,* respectively. Novel nucleotide sequences for *selx8-11* and *selxbov2* have been deposited in GenBank under accession numbers HQ850970, HQ850971, HQ850968, HQ850969 and HQ85097, respectively. Of note, the human ST398 strain OS385 contains *selx13*, which is a 315 bp truncated C-terminal fragment of the gene predicted to be non-functional. With the exception of the truncated *selx13* there are 85.9% non-variable nucleotide sites among the *selx* alleles identified. This corresponds to a total of 40 (13%) variable amino acid residues among the 204 amino acid sites of SElX (Fig. S2). Pairwise amino acid alignment reveals 89.5 to 98.5% amino acid identity among the different allelic variants made by the major *S. aureus* pathogenic clones (Fig. S2).

In general, each clonal complex (consisting of closely-related STs) contains a unique *selx* allele (Fig. 1b) consistent with point mutation being the major driving force for evolution within individual lineages. However, allele *selx2* was identified in ST12, ST15 and CC8 strains, and *selx5* was encoded by both ST1 and ST72 strains, respectively, indicating that assortative recombination of the *selx* gene has contributed to its distribution among some lineages which have not shared a recent common ancestor (Fig. 1b). In addition, examination of the contribution of recombination to *selx* diversity employing the RDP3 suite of programs [23], revealed evidence for at least 3 distinct recombination events leading to hybrid alleles of *selx* (Fig. S3). Furthermore, a phylogenetic tree based on *selx* gene sequences had a topology distinct from the phylogenetic tree derived from concatenated MLST loci (Fig. S4). Taken together, these data suggest that recombination has contributed to the diversification and distribution of *selx* among *S. aureus* clones.

### SElX is expressed by clinical isolates *in vitro* and during human, bovine and ovine infection

**Figure 2:**
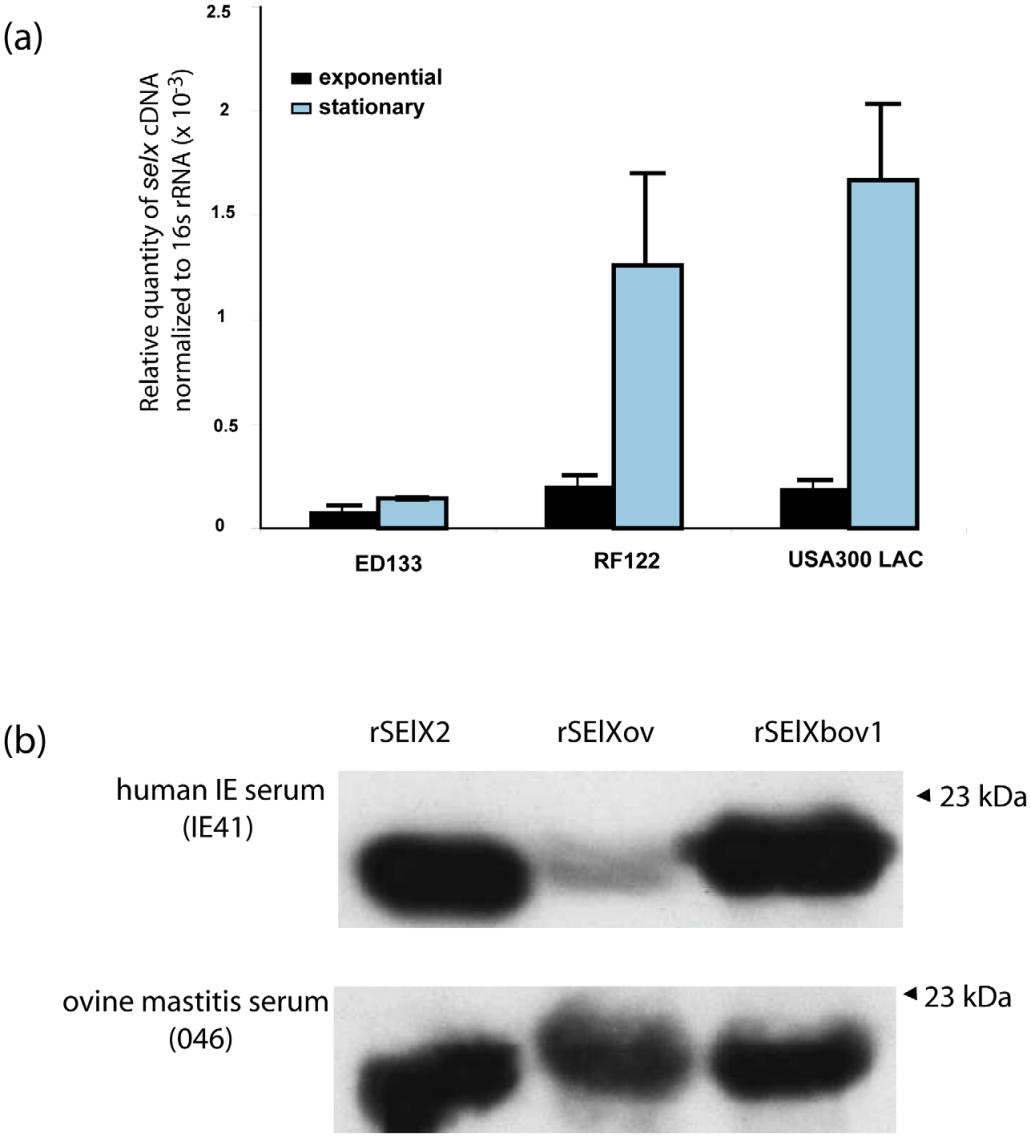
SElX is expressed by clinical isolates *in vitro* and during infection. **a**) Growth-phase dependent transcription of *selx* determined by qRT-PCR. **b**). Representative Western blot analysis of rSElX proteins with human infective endocarditis (IE) or ovine mastitis sera samples.

In order to determine whether *selx* was expressed by clinical isolates we examined by quantitative real-time PCR (qRT-PCR), the transcription of *selx* by human *S. aureus* CA-MRSA USA300 strain LAC, bovine strain RF122, and ovine strain ED133 during exponential and stationary phases of growth *in vitro*. *selx* was transcribed by each strain in a growth-phase dependent manner, and the highest relative expression level was demonstrated by USA300 strain LAC (Fig. 2). To investigate the *in vitro* expression of SElX we carried out Western blot analysis with stationary phase culture supernates of 15 representative clinical isolates of *S. aureus* of human, bovine, and ovine origin, with rat anti-serum specific for SElX. Expression of SElX was detected in 13 of 15 isolates including 5 of 5 human isolates from sepsis, scalded skin syndrome and infective endocarditis patients, 4 of 4 bovine mastitis isolates, 3 of 4 ovine mastitis isolates, and a single caprine mastitis isolate. A single avian isolate ED98 did not express SElX in detectable quantities. These data indicate that SElX is made by the majority of clinical isolates at detectable levels in nutrient replete conditions *in vitro* (Table S3).

To determine whether SElX is expressed during human and animal colonization or infection, Western immunoblot analysis was carried out using recombinant SElX human (SElX2), bovine (SElXbov1) and ovine (SElXov) variants, with sera from humans, cows and sheep recovering from *S. aureus* infections, and from healthy human volunteers. Specific SElX antisera did not cross-react with TSST-1 or SSL7, its closest homologs (Fig. S5). In addition, we identified individuals who were seroconverted for SElX but not for SSL7 or TSST-1, and an individual who was seroconverted for SSL7 and TSST-1 but not for SElX (Fig. S5). Taken together, these data indicate a lack of cross-reactivity of antibodies specific for TSST-1, SSL7 and SElX. All 5 human, 4 bovine and 5 ovine serum samples from infected individuals, and 20 of 23 serum samples from healthy humans contained antibody that recognized the SElX recombinant proteins, whereas only one serum sample (from a healthy human) out of the total of 47 contained antibody that recognized recombinant SElO SAg (Table 1). These data indicate that SElX is made during *S. aureus* colonization or infection of humans and animals and stimulates a humoral immune response. Of note, densitometric analysis of SElX immuno-reactive bands indicated approximately 2-fold lower reactivity of human IgG with recombinant SElXov, in comparison to IgG from *S. aureus*-infected sheep, suggesting that SElXov made by ovine *S. aureus* may vary antigenically (Fig. 2 b). Overall, the expression of SElX during infection of humans and animals suggests an important general role during *S. aureus* infection of multiple host species.

**Table 1:** Immunoreactivity of recombinant SAg variants with sera from healthy humans, and bovine, ovine and human patients recovering from *S. aureus* infections.

Serum sample	SElXbov1 a	SElXov a	SElX2 a	SElO a
**Human (Normal)**				
HV093	n/d	n/d	+	-
HVRC	n/d	n/d	-	+
HVJRF	n/d	n/d	-	-
HV100	n/d	n/d	-	-
HV091	n/d	n/d	+	-
HV126	n/d	n/d	+	-
HV097	n/d	n/d	+	-
HV101	n/d	n/d	+	-
HV118	n/d	n/d	+	-
HV115	n/d	n/d	+	-
HV017	n/d	n/d	+	-
HV105	n/d	n/d	+	-
HV003	n/d	n/d	+	-
HV008	n/d	n/d	+	-
HV148	n/d	n/d	+	-
HV139	n/d	n/d	+	-
HV014	n/d	n/d	+	-
HV047	n/d	n/d	+	-
HV020	n/d	n/d	+	-
HV057	n/d	n/d	+	-
HV104	n/d	n/d	+	-
HV002	n/d	n/d	+	-
HV098	n/d	n/d	+	-
**Human IE**				
IE19	+	+	+	-
IE37	+	+	+	-
IE41	+	+	+	-

Serum sample	SElXbov1 a	SElXov a	SElX2 a	SElO a
IE51	+	+	+	-
IE54	+	+	+	-
**Bovine mastitis**				
2480	+	+	+	-
2487	+	+	+	-
2521	+	+	+	-
4227	+	+	+	-
2211	-	-	-	-
**Ovine mastitis**				
011	+	+	+	-
040	+	+	+	-
046	+	+	+	-
0112	+	+	+	-
0348	+	+	+	-

a **+,** positive reactivity; -, negative reactivity; n/d, not done.

### SElX has a unique predicted SAg structure

**Figure 3:**
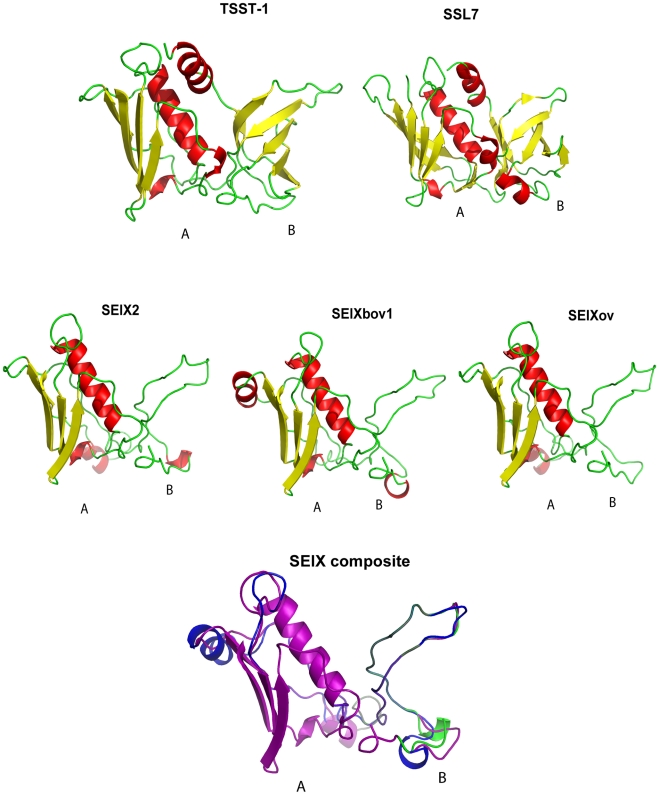
SElX is predicted to have a truncated B-domain. Schematic diagram of the solved structures of TSST-1, SSL7 and modelled 2-domain structures of human, bovine and ovine variants of SElX. SElX composite structure was obtained by superimposition of protein variants in PyMol.

The SElX2 variant encoded by *S. aureus* USA300 strain LAC, contains 27% and 24% amino acid identity with its nearest protein homologs, superantigen-like protein 7 (SSL7) and TSST-1, respectively. In order to investigate the predicted structure of the novel SAg, we carried out structural modeling using the program Phyre [24] with SSL7 and TSST-1 crystal structures from the PDB database as templates. Considering, the low sequence homology of SElX with its closest homologs SSL7 and TSST-1, used as templates for modeling, the results should be considered to be speculative. The hypothetical predicted structures of SElX variants imply a potential to form a characteristic 2 domain SAg structure with some unusual features (Fig. 3). The predicted A-domain containing the C-terminus has a long central α-helix, positioned along one side of a 4-stranded β-sheet, forming a structure typical of the β-grasp motif [25]. However the B-domain which contains the N-terminus, is considerably smaller than the B-domain of previously characterized SAgs and lacks an OB fold (Fig. 3). The OB fold is the site typically involved in SAg binding to the α-chain of the MHC class II complex on antigen presenting cells [2], [26]. The functional implications of this unique predicted B-domain structure are currently unknown.

### SElX is mitogenic and demonstrates Vβ-dependent T-cell proliferation

**Figure 4:**
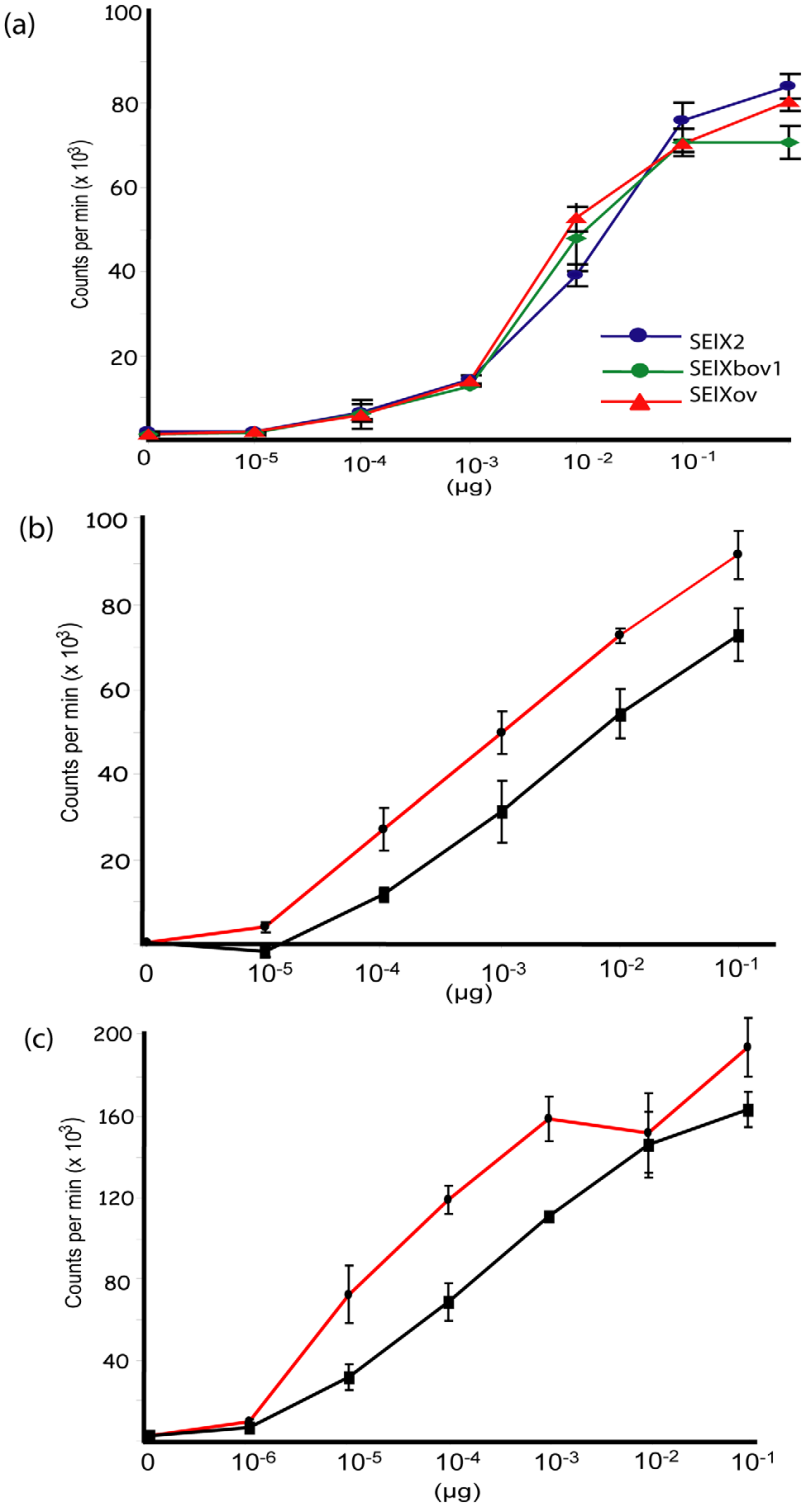
SElX stimulates proliferation of human and rabbit lymphocytes. **a**) Proliferation of human PBMC cultures with recombinant SElX2, SElXbov1, and SElXov variants measured by [3H]-thymidine incorporation. Proliferation of human (b) or rabbit (c) PBMC cultures with recombinant SElX2 (red line) or TSST-1 (black line) measured by [3H]-thymidine incorporation.

We examined the function of representative allelic variants of SElX from human, bovine and ovine isolates. Recombinant human (rSElX2), bovine (rSElXbov1), and ovine (rSElXov) variants were purified and used to stimulate human PBMC in a thymidine incorporation assay. All 3 rSElX variants were mitogenic for human T-cells at concentrations above 0.1 ng/µl (Fig. 4a). Further, in order to examine the mitogenicity of SElX expressed in a native *S. aureus* background, *selxbov1* was cloned into the pALC2073 vector and expressed in a SAg-deficient strain RF122-8. The supernatant from cultures of RF122-8 + pALC2073:SElXbov1 had mitogenic activity for bovine lymphocytes, but supernatant from strain RF122-8 containing the pALC2073 plasmid only had none (data not shown). SElX was also mitogenic for rabbit lymphocytes and importantly, the superantigenic activity of SElX for both human and rabbit T-cells is comparable or higher to the activity of TSST-1, the SAg responsible for the toxinoses toxic shock syndrome (TSS) (Fig. 4b, c).

**Figure 5:**
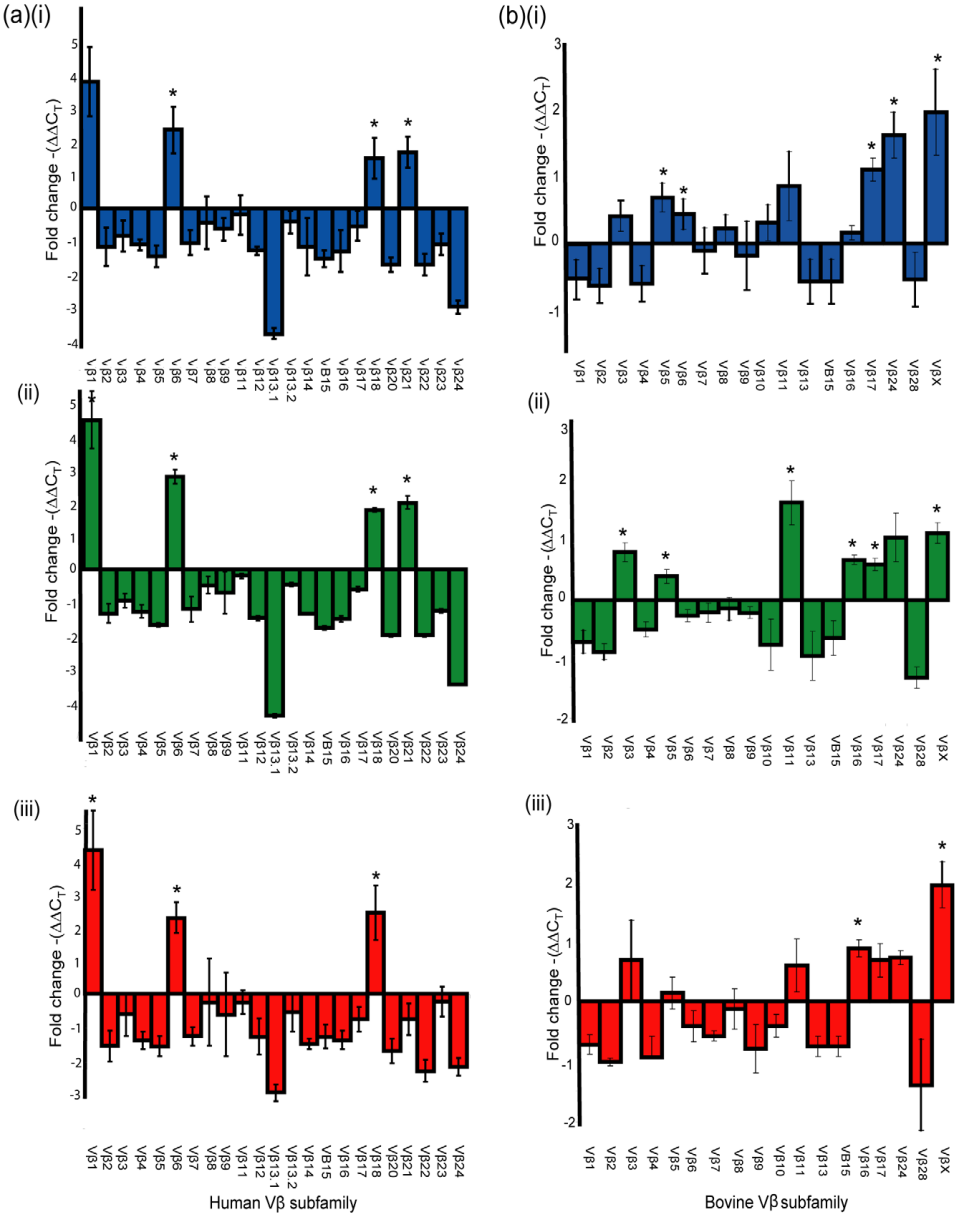
SElX activates Vβ-specific human and bovine Tcells. (ii) Relative fold-change in Vβ expression (mean ± S.E.M.) for human (A) or bovine (B) T cells after stimulation with (i) SElX2_,_ (ii) SElXbov1, and (iii) SElXov. *indicates statistical significance (p<0.05).

We recently designed a novel assay for quantifying human Vβ (humVβ) gene expression including all of the known Vβ subgroups [27]. Previous studies of the bovine Vβ (bovVβ)-dependent expansion capacity of staphylococcal SAgs have been restricted by the number of identified bovine Vβ subfamilies [11], [28]. The recent bovine genome sequencing project has facilitated a comprehensive description of the bovine Vβ subgroups and the repertoire of functional Vβ genes [29], [30]. In the current study, design of a novel bovVβ-dependent expansion assay has allowed us for the first time to evaluate the response of 23 human and 18 bovine Vβ subfamilies to stimulation with a staphylococcal SAg (Fig. 5a, b). rSElX2 and rSElXbov1 activated humVβ subfamiles 1, 6, 18 and 21, whereas rSElXov activated 1, 6 and 18 but not 21 (Table 2). All SElX variants stimulated bovVβ subfamily X. In addition SElXov activated bovVβ 16, SElXbov1 activated bovVβ 3, 5, 11, 16 and 17, and SElX2 activated bovVβ 5, 6, 17 and 24. Different human and bovine Vβ subfamilies are activated in response to stimulation with SElX. This is in part due to the activation of humVβ subfamilies 18 and 21, for which there are no bovine orthologs, and bovVβ X which has no human ortholog. However, the orthologous humVβ 6 and bovVβ 6 are both activated by SElX2. In addition, bovVβ subfamily 16 which is activated by SElXbov1 and SElXov is phylogenetically related to humVβ 6. Of note, the humVβ 1 subfamily and the closely related bovVβ 5 but not the orthologous bovVβ 1 were activated by SElX variants. These data indicate a unique pattern of Vβ gene activation for SElX in comparison to other previously characterized SAgs [3], [27]. Importantly, the results demonstrate differences in superantigenic activity and distinct Vβ activation profiles for different SElX variants made by *S. aureus* strains associated with different host species.

**Table 2:** Human and bovine Vβ subfamilies activated in response to rSElX variants.

SElX variant	Human Vβ a, b	Bovine Vβ a, b
SElX2	**1, 6,** **18,** **21**	3, **5, 6**, 11, **17**, **24**, **X**
SElXbov1	**1, 6, 18,** **21**	**3, 5, 11, 16, 17,** 24**, X**
SElXov	**1, 6, 18**	**16**, 17, 24, **X**

a Vβ subfamily nomenclature followed the classification of Arden *et al* [65].

b Bold type indicates subfamilies activated with a significance of p<0.05, and normal type indicates subfamilies with a trend (p<0.1) towards significant activation above baseline control (unstimulated).

### SElX has functional activities characteristic of SAgs

**Figure 6:**
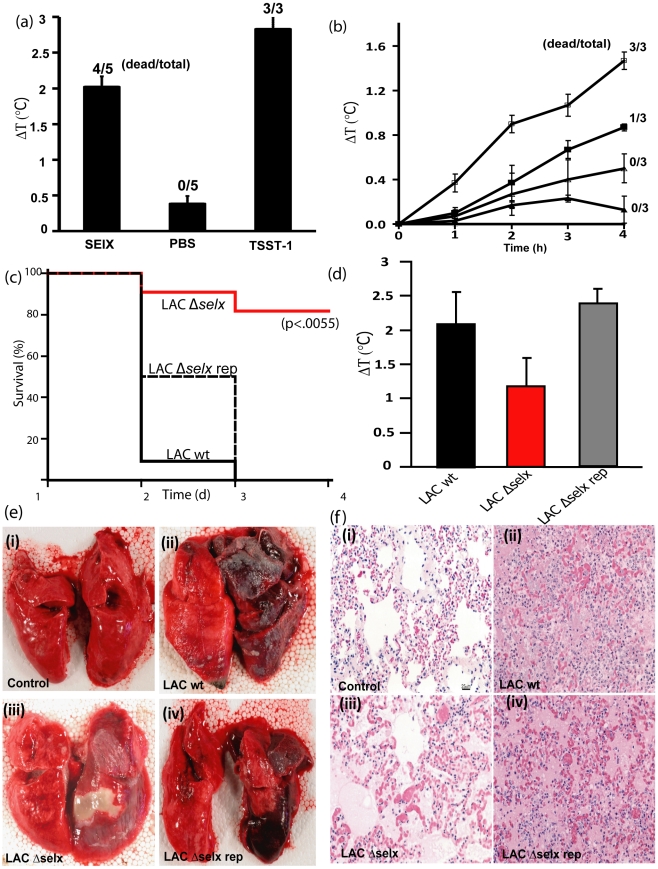
SElX causes TSS and contributes to the severity and lethality of necrotizing pneumonia caused by CA-MRSA USA300. **A**) The ability of rSElX2, administered at a dose of 200 µg/kg, to cause TSS was measured using a standard miniosmotic pump model of TSS. TSST-1 administration was used as the positive control and PBS the negative control. **B**) Fever in response to intravenous injection of rSElX2 at concentrations of 10 µg/kg (□), 1 µg/kg (▪), 0.1 µg/kg (Δ), of body weight per ml, or PBS (▴), was evaluated over a 4 h test period, followed by the ability to enhance the lethality of LPS over 48 h. **C**) Kaplan-Meier Curves of % survival of rabbits infected with *S. aureus* LAC, *S. aureus* LACΔ*selx*, and LACΔ*selx* rep. **D**). Increase in rabbit core temperature (ΔT °C) 2 d after pulmonary infection with LAC wt, LACΔ*selx*, and LACΔ*selx* rep. **E**). Gross pathology of lungs from uninfected rabbit (i) or from rabbits infected with LAC wt (ii), LACΔ*selx* (iii), and LACΔ*selx* rep (iv). **F**) Haematoxylin Eosin-stained tissue sections from (i) uninfected rabbit lung, or from rabbit lungs infected with (ii) LAC wt, (iii) LACΔ*selx* and (iv) LACΔ*selx* rep isogenic strains.

We examined the ability of rSElX2 to cause TSS in rabbits using a standard mini-osmotic pump model of TSS at a dose of 200 µg/kg. Of the 5 rabbits given rSElX2, 4 succumbed within 7 d, compared to 0 of 5 treated with PBS (p<0.05) (Fig. 6a). The positive control TSST-1 was lethal for 3 of 3 animals in the 7 d test period (Fig. 6a). Changes in temperature of the rabbits were recorded before and 24 h post-SAg challenge. Rabbits treated with rSElX2 developed fever with a 2.0°C increase in body temperature over the 24 h test period, compared to 0.4°C for PBS treated animals (p<0.001) (Fig. 6a).

Rabbits were also used to evaluate fever responses over 4 h after intravenous injection, followed by the ability to enhance lethality of LPS over a 48 h test period. The capacity to cause fever that peaks 4 h after injection, and to amplify the lethal effects of LPS by up to 10^6^-fold are defining activities of SAgs. rSElX2 caused dose-dependent fever responses in rabbits that peaked 4 h after injection and the 4 h fever response of animals receiving 10 µg/kg of rSElX2, compared to PBS, was significantly different (p value of 0.001) (Fig. 6b). The minimum pyrogenic dose of SAg is defined as the dose per kg required to cause an average 0.5°C rise in rabbit body temperature in 3 animals; the minimum pyrogenic dose of rSElX2 was calculated to be 1.0 µg/kg. rSElX2 also enhanced rabbit susceptibility to lethal shock by LPS (Fig. 6b). Taken together, rSElX2 demonstrated the functional characteristics required for classification as a SAg, including mitogenicity, pyrogenicity, enhancement of endotoxin shock, and lethality when administered by mini-osmotic pump.

### SElX contributes to lethality of CA MRSA USA300 in a rabbit model of necrotizing pneumonia

CA-MRSA is notorious for causing skin and soft tissue infections, and severe necrotizing pneumonia [20], [31]. We found that CA-MRSA USA300 strains expressed elevated levels of *selx in vitro* relative to other clinical isolates (Fig. 2a). In order to investigate the hypothesis that SElX contributes to the pathogenesis of necrotizing pneumonia, we constructed an SElX-deficient mutant of CA-MRSA USA300 strain LAC (LAC Δ*selx*), and a repaired derivative with an intact functional *selx* gene (LAC Δ*selx* rep). To rule out the possibility of spurious mutations acquired during the construction of LAC Δ*selx* in loci which could influence virulence, such as the accessory gene regulator (*agr)* [32], we compared the isogenic LAC wild type, LAC Δ*selx* and LAC Δ*selx* rep strains for hemolytic titre, secreted and cell wall-associated protein profiles, and *in vitro* growth rate. In each case we found that the strains were indistinguishable (Fig. S6 and data not shown). In addition, we compared LAC wild type and LAC Δ*selx* for α-toxin and PVL expression levels by ELISA and found identical levels of expression (data not shown). We then compared the ability of wild type, mutant and repaired strains to cause lethal necrotizing pneumonia in a rabbit model [33]. For animals receiving wild type LAC, all 11 rabbits succumbed within 4 d, compared to 2 of 11 receiving LAC Δ*selx* (p<0.002) (Fig. 6c). Importantly, 4 of 4 rabbits infected with the repaired strain LAC Δ*selx* rep succumbed within 4 d (Fig. 6c). Body temperatures were recorded in the first 24 h of the experiment, and animals receiving strain LAC demonstrated significantly higher body temperatures than animals receiving the SElX-deficient strain LAC Δ*selx* (p<0.002) and LAC Δ*selx* rep had wild type levels of pyrogenicity (Fig. 6d). On gross examination, regionally extensive to lobar areas of lung from rabbits infected with wild type USA300 LAC and the repaired strain USA300 LAC Δ*selx* rep were dark red to purple, heavy and oozed blood on the cut surface (Fig. 6e). In contrast, lungs from rabbits infected with the LAC Δ*selx* mutant strain were reddened with well demarcated, focal white areas (abscess), but did not show signs of haemorrhage (Fig. 6e). Histological examination of tissues from both the wild type USA300 LAC and the repaired strain USA300 LAC Δ*selx* rep revealed severe haemorrhagic and necrotising pneumonia (Fig. 6f). Tissue sections from rabbits infected with the LAC Δ*selx* mutant strain were only moderately affected (Fig. 6f). Taken together, these data collectively indicate that SElX made by USA300 LAC causes high fever and contributes to severity of infection and lethality in a rabbit model of necrotizing pneumonia.

## Discussion

We have identified a novel SAg which is encoded by the great majority of strains, and which causes lethality in a model of severe *S. aureus* human disease. The distribution of *selx* across the full breadth of *S. aureus* diversity, its absence among other staphylococcal species, and its genetic linkage with an integrase pseudogene suggests an ancient horizontal acquisition event which happened prior to the most recent common ancestor of the *S. aureus* species. The gene is absent from only a single clone examined (CC30) but the associated integrase pseudogene is retained suggesting that a deletion event has resulted in the loss of *selx* during the evolution of the CC30 lineage. Of note, previous studies have discovered that the majority of CC30 isolates contain a SaPI which encodes TSST-1, the most closely-related SAg to SElX [34], [35]. These data imply that virtually all *S. aureus* isolates have the capacity to produce either TSST-1 or SElX suggesting an important role for this sub-group of toxins in *S. aureus* pathogenesis.

The *selx* gene has undergone diversification leading to at least 17 different allelic variants identified among the major clonal lineages (Fig. 1 b). Although SElX is the first SAg identified which is encoded in the core genome of *S. aureus*, the SAgs, SMEZ and SPEG, are made by the majority of Group A Streptococcal (GAS) isolates [36]. Extensive allelic variation of SMEZ contributes to antigenic variation, but does not influence Vβ-specificity or mitogenicity [36]. Whereas GAS is specific for human hosts, *S. aureus* is also represented by strains which are specialized for ruminant or avian host species [37], [38]. In contrast to SMEZ, SElX bovine- and ovine-specific variants displayed distinct Vβ subgroup proliferation profiles for bovine lymphocytes in comparison to a human-specific SElX variant suggesting that they have undergone adaptive diversification leading to enhanced activity in ruminants. Previously, it was shown that the proliferation of human and bovine lymphocytes varied in response to stimulation with allelic variants of the SAg staphylococcal enterotoxin C (SEC). The SEC bovine variant (SECbov) requires between 10- and 1000-fold more toxin than SEC1 and the SEC ovine variant (SECov) to induce proliferation of human or bovine PBMCs, and results in a distinct Vβ-dependent T-cell activation profile [28], [39].

Although most of the allelic variation in *selx* was clonal and due to point mutation, we found evidence of a role for recombination in the diversification and distribution of SElX, including whole gene transfer of the *selx* allele between pathogenic clones. Recombination rates are predicted to be low in *S. aureus* in general [40], but some recombination, particularly among genes involved in virulence has been observed [41], [42]. Such rapid evolution may facilitate antigenic or functional diversification of proteins which are critical for bacterial survival during infection.

Analysis of *selx* transcription levels revealed a growth phase-dependent expression analogous to numerous other staphylococcal virulence proteins which are under the control of *agr*. Of note, the human CA-MRSA USA300 strain LAC had relatively high levels of expression of SElX which correlates with the previously reported up-regulation of RNAIII and secreted virulence proteins by the USA300 epidemic clone [43]. In addition to demonstrating the *in vitro* expression of SElX by *S. aureus* strains, we also identified SElX-specific antibodies in human and ruminant convalescent serum samples, and in healthy individuals. The high frequency of sero-conversion to SElX among human and livestock populations is consistent with the expression of SElX by most commensal and disease-associated isolates of *S. aureus,* and imply a broad role for SElX in *S. aureus* colonization or infection of multiple host species.

Of particular importance, the USA300 epidemic clone is currently the primary infectious cause of human mortality in the USA, and unlike most strains of hospital-associated MRSA can cause disease in otherwise healthy individuals [44]. The molecular basis for the increased virulence of USA300 strains has been the subject of intensive research efforts. Several reports have highlighted the high levels of expression of secreted proteins such as Panton-Valentine leukocidin, α-toxin, and phenol-soluble modulins and evidence exists for a role for each of these toxins in the pathogenesis of CA-MRSA severe infection [43], [45]–[50]. Recently, Assimacopoulos *et al.* described an *S. aureus* extreme pyrexia syndrome that is associated with CA-MRSA USA300 strains [51]. Our studies suggest that SElX contributes to the pathogenesis of pulmonary illness caused by these strains, and its induction of high fever during infection is consistent with a role in extreme pyrexia syndrome. While most studies on the pathogenesis of severe *S. aureus* pulmonary infections have focused on the role of cytolysins [43], [45], [49], [50], our data suggest that the novel SAg SElX may influence the outcome of severe infection caused by CA-MRSA USA300. This is in agreement with a recent study that shows that TSST-1, SEB and SEC contribute to lethal pneumonia of rabbits caused by CA-MRSA USA200 and USA400 strains [33]. Of note, rabbits are much more similar to humans than mice in their sensitivity to both SAgs and cytolytic toxins [52]. Importantly, we have shown that SElX is mitogenic for both rabbit and human T cells. As the majority of previous studies into the virulence of *S. aureus* infections have been carried out in mice, the importance of SAgs in the pathogenesis of severe infections may have been underestimated.

Because of the role of SAgs in *S. aureus* pathogenesis and their potential as an agent of biological warfare, therapeutic strategies which target these toxins have been the focus of considerable research efforts [6]. Structural modeling revealed the potential of SElX to form the characteristic 2 domain SAg structure joined by a central α-helix. However domain B of SElX is predicted to be much smaller than that of other SAgs identified to date (Fig. 4). Ongoing crystallography analyses should result in important insights into the molecular interaction of the uniquely structured SElX with its ligands which may facilitate the design of molecules to inhibit its function.

CA-MRSA USA300 strains are characterized by high expression of a number of secreted virulence factors which through direct activity or immunopathology result in the severe symptoms associated with infections such as necrotizing pneumonia [43], [45]–[50]. Here, we report the discovery of a novel secreted virulence determinant made by most strains of *S. aureus* that may contribute to the severity of some human diseases caused by highly-virulent clones such as CA-MRSA USA300. Furthermore, the phylogenetic and immunobiological characterization of a unique *S. aureus* core genome-encoded SAg provides new insights into the evolution of pathogenic *S. aureus* and its capacity to cause disease in multiple host species.

## Materials and Methods

### Ethics statement

All animals were handled in strict accordance with good animal practice as defined by the relevant national and/or local animal welfare bodies. Animal experimentation was performed under a University of Minnesota approved Institutional Animal Care and Use Committee (IACUC) protocol (0908A71722). University of Minnesota is accredited by the Association for Assessment and Accreditation of Laboratory Animal Care International (AALAC). Animals are maintained in accordance with the applicable portions of the Animal Welfare Act and the DHHS “Guide for the Care and Use of Laboratory Animals”. In agreement with the University of Minnesota IACUC, animals that failed to exhibit escape behaviour and at the same time could not right themselves were prematurely euthanized. Animals were euthanized with intravenous injection of 1 ml of Beuthanasia D, whether prematurely or at the end of experimentation. Human peripheral blood mononuclear cells were isolated from heparinized venous blood of four different healthy donors in accordance with a human subject protocol approved by the University of Idaho Institutional Review Board for Human Subjects (approval number 05–056), in accordance with the principles of the Declaration of Helsinki. Donors were informed the procedure risks and provided a written consent prior to participation. The University of Idaho is fully accredited by the AALAC.

### S. aureus strains

*S. aureus* strains were selected to represent clonal genotypes which represent the breadth of species diversity, different host-associations and geographic origins (Table S3). *S. aureus* strains were grown in tryptone soya broth (TSB) or brain heart infusion (BHI) broth with shaking at 200 rpm, or on tryptone soya agar (TSA) plates. For use in animal studies, organisms were cultured in Todd Hewitt broth or on Todd Hewitt agar. Media was supplemented where appropriate with chloramphenicol (Sigma-Aldrich) at 12.5 µg/ml and 150 µg/ml X-gal (5-bromo-4-chloro-3-indolyl-beta-D-galactopyranoside) (Melford).

### Bioinformatic analyses

The novel putative SAg gene, *selx* was first identified by BLAST analysis of the genome of USA300 FPR3757 with the gene sequence specific for the SAg TSST-1. The sequences of distinct *selx* alleles were identified by BLASTn analysis of *S. aureus* whole genome sequences deposited in GenBank (Table S1). Additional *selx* alleles were sequenced with *selx*seq primers (Table S2) by Genepool Sequencing Service (University of Edinburgh, UK), aligned by ClustalW using MEGA 4.0.1 software [53] and Neighbor-Joining phylogenetic trees constructed using the Nucleotide Maximum Composite Likelihood model. At least 500 bootstrap trees were generated to examine the robustness of the inferred phylogenetic relationships. Recombination detection program RDP v3.44 was used to identify putative end points of recombination events [23]. Unique recombination events were detected by at least 3 of the programs employed by the RDP suite, p<0.05. Protein structural modeling of the derived amino acid sequences of *selx2, selxbov1* and *selxov* was carried out. Predicted 3D structures were obtained for SElX2, SElXbov1 and SElXov with the program Phyre using the crystal structure of TSST-1 as a template, (PDB file c5tssA) [24]. PDB files were generated for each predicted structure and analyzed using Pymol software [54]. Amino acid sequence alignment was carried out using ClustalW and viewed with Jalview [55].

### Transcriptional analysis of *selx*

Total RNA was extracted from *S. aureus* strains LAC, RF122, and ED133 exponential (OD_600_ = 0.6) and stationary phase (16 h) cultures using the RNeasy miniprep kit (QIAgen) as described in the manufacturer's instructions except for re-suspension in TE buffer with 100 µg/ml Lysostaphin and incubation at 37°C for 20 min. RNA was treated with Turbo DNase (Ambion Inc) and. 0.5 µg mRNA from at least 3 independent total RNA extractions were reverse-transcribed to cDNA with the Power SYBR Green RNA-to-CT 2-Step Kit (Applied Biosystems). To quantify cDNA generated by reverse transcription from target RNA, qRT-PCR reactions were carried out in 25 µl reactions containing 50 ng of cDNA, 300 nM *selxq* or *16S rRNA* primers (Table S2), and SYBR Green I dye master mix (AB), using a Mx3000P light cycler (Strategene). *16S rRNA* primers have been described elsewhere [56]. Relative values of transcription of *selx* were determined by comparative quantification to the internal control *16SrRNA*. The thermal conditions were: 10 min at 95°C for 1 cycle, 20 s at 95°C, 20 s at 60°C and 20 s at 72°C for 40 cycles. RNA samples were processed in triplicate with no template (NTC), no Reverse Transcriptase (no RT) and positive genomic DNA controls. Fluorescence was measured at the end of the annealing phase of each cycle and a threshold value for the fluorescence set by the MxPro qPCR software version 4.1.

### Cloning and purification of recombinant SAgs

5′ primers for cloning *selx2*, *selxbov1*, and *selxov* into the pET15b plasmid (Novagen), were designed to anneal immediately after the signal peptide coding region, as predicted by Signal P 3.0 Server (http://www.cbs.dtu.dk/services/SignalP/), and 3′ primers were designed to include the stop codon of the gene (Table S2). PCR reactions were carried out using Vent polymerase (NEB) and contained 100 nM *selx*pET forward and reverse primers designed to incorporate restriction sites *Nde*I and *Bam*HI (Table S2) and 10 ng of template DNA. PCR products were restriction digested with *Nde*I and *Bam*HI (NEB), purified, and ligated with T4 DNA ligase (NEB), and transformed into *E. coli* DH5α cells. pET constructs were isolated from DH5α using the QIAprep Spin Miniprep kit (QIAgen), and transformed into *E. coli* BL21. BL21 cells containing the pET plasmid constructs were cultured in Luria-broth containing 50 µg/ml ampicillin (Sigma-Aldrich) and induced in mid-exponential phase of growth (OD_600_ = 0.6), with 1 mM isopropyl β-D-1-thiogalactopyranoside (IPTG) (ForMedium Ltd.) for 4 h. Cells were recovered by centrifugation at 8000 xg, disrupted using a French Press, and His-tagged recombinant proteins were purified by affinity chromatography on a Ni-NTA nickel affinity column (Invitrogen). Proteins were dialysed using Spectra/Por Float-A-Lyzer tubing with a 8000 to 10000 molecular weight cut off (MWCO) (Spectrum Laboratories), and LPS was removed using ProteoSpin endotoxin removal kit (Norgen Biotek).

### Western immunoblot analysis

Supernates from stationary phase cultures of *S. aureus* strains were concentrated with Amicon Ultra-15 Centrifugal Filter units (10000 MWCO) (Millipore). Recombinant proteins and concentrated secreted proteins were separated by sodium dodecyl sulphate-polyacrylamide gel electrophoresis (SDS-PAGE) and transferred to nitrocellulose membranes (Amersham Hybond ECL, GE Healthcare) in Towbin transfer buffer. The membrane was incubated in 1 x PBS (pH 7.3) containing 8% powdered milk (Fluka), at 4°C overnight, washed 3 times with washing buffer, 1 x PBS (pH 7.3) containing 1% powdered milk and 0.05% Tween 20 (Sigma-Aldrich). The membrane was incubated for 1 h with primary antibody, which was either a 1∶2500 (bovine and ovine) or 1∶5000 (human) dilution of serum, or 2 h with 1∶1500 dilution of rat anti-sera raised against rSElXbov1. The membrane was then incubated with horse radish perixodase conjugated (HRP) secondary antibodies for 1 h, which was 1∶2500 goat anti-bovine IgG, 1∶2500 rabbit anti-sheep IgG, (Santa Cruz Biotechnology), 1∶5000 rabbit anti-human IgG (Dako), or 1∶1500 goat anti- rat IgG (Abcam), depending on the experiment. Human sera samples were obtained from infectious endocarditis patients [57], ovine serum samples were obtained from experimentally-infected sheep and provided by E. Vautor [58] and from cows with bovine mastitis by C. Smyth. Semi-quantitative spot densitometry was carried out with the ChemiImager 4000i.V4 program, using a MultiImager light cabinet (Alpha Innotech).

### Construction of *selx*-deficient and *selx*-repaired derivatives of *S. aureus* USA300 LAC

An *selx*-deficient strain of USA300 LAC was obtained by allele replacement using the pMAD-CM plasmid [59]. PCR products of 578 bp and 592 bp, flanking the left (AB) and right (CD) regions of *selx*, respectively were amplified with primers listed in Table S2. PCR amplification with primers A and D was carried out with 100 ng of purified AB and CD template DNA to produce a single spliced AD fragment by overlapping PCR, facilitated by sequence complementary to primer B incorporated into the CD fragment by primer C. AD products were then purified and cloned into the Strataclone pSC-B plasmid (Stratagene). pSC-B plasmid containing the AD insert was digested with *Eco*RI for 3 h at 37°C. The AD fragment was then purified, ligated into the dephosphorylated gene replacement plasmid, pMAD-CM , to create pMAD::*selx*, which was transformed by electroporation into LAC. LAC containing pMAD::*selx* was shaken overnight at 30°C in TSB containing chloramphenicol. To select for integration of the plasmid into the chromosome through homologous recombination, 10-fold dilutions were incubated on TSA containing chloramphenicol at 44°C, a temperature non-permissive for plasmid replication. Double cross-over excisants were selected for by growing integrants in TSB without chloramphenicol at 30°C for 24 h. 10-fold dilutions were plated onto TSA containing X-gal and incubated on TSA at 30°C overnight. White colonies were screened for antibiotic sensitivity on chloramphenicol plates (inferring loss of the plasmid) and screened for WT or mutated forms of the gene by PCR with primers upstream (E) and downstream (Z) of *selx* (Table S2). The resulting *selx-*deficient strain, LACΔ*selx* was sequenced using E and Z primers in order to confirm the in-frame deletion event. The *selx* deletion in LACΔ*selx* was repaired using an identical allelic exchange approach employing pMAD-CM. Oligonucleotide primers (Table S2) designed to incorporate a single synonymous substitution into the *selx* gene were used to amplify 2 PCR products specific for left and right regions of the *selx* gene (rep AB and rep CD primers). The resulting PCR products were spliced to produce an intact *selx* gene, and cloned into pMAD as previously described resulting in pMAD::*selx* rep. Allelic exchange was carried out as described for *selx* mutant construction and the repaired *selx* gene sequenced. Western blot analysis confirmed the restoration of SElX expression (Fig. S6a).

### Pyrogenicity, endotoxin enhancement and miniosmotic pump lethality studies

American Dutch Belted rabbits were injected with rSElX2 at doses of 10, 1, and 0.1 µg/kg of body weight per ml intravenously. Three rabbits were injected with each dose and temperature was measured hourly for 4 h. After 4 h, each rabbit was injected intravenously with 1 µg of lipopolysaccharide (LPS) from *Salmonella enterica* serovar typhimurium (1/500 of the 50% lethal dose of endotoxin alone). SAgs characteristically amplify the lethal effects of LPS by up to 10^6^-fold. Lethality was assessed over a 48 h period [60]. In agreement with the University of Minnesota IACUC, animals that failed to exhibit escape behaviour and at the same time could not right themselves were prematurely euthanized. It is our experience in over 30 years of similar experimentation that this point is 100% predictive of SAg lethality. Animals were euthanized with intravenous injection of 1 ml of Beuthanasia D, whether prematurely or at the end of experimentation. Miniosmotic pumps, containing 200 µg of rSElX2 or TSST-1, or PBS, were implanted subcutaneously into 5 American Dutch Belted rabbits per group (3 for TSST-1) [61]. Fever was assessed after 24 h, and lethality of the toxins over a period of 7 d. The same criteria as above were used for assessing need for premature euthanasia.

### Necrotizing pneumonia rabbit model

Wild-type LAC and the *selx* knock-out strain were cultured in Todd Hewitt broth for 16 h and washed once in Todd Hewitt broth to remove exoproteins. The bacteria were re-suspended in Todd Hewitt broth at 1×10^10^ colony-forming units (CFU)/ml for use in injections. American Dutch Belted rabbits (11 per group for LAC wt and LAC Δ*selx*, and 4 for LAC Δ*selx* rep) were anesthetized with ketamine and xylazine. Their tracheas were exposed and 2×10^9^ USA300 CA-MRSA strain LAC, the isogenic *selx-*deficient LAC strain or its repaired derivative were administered intra-tracheally through catheters in 0.2 ml volumes. The animals were closed and monitored for 4 d for development of fatal necrotizing pneumonia.

### Preparation and stimulation of lymphocytes

Blood was obtained from 2 Holstein–Friesian cattle aged 18–36 m via jugular vein puncture. Animals were reared indoors and maintained on a ration of hay and concentrates. Peripheral blood mononuclear cells (PBMC) were isolated from blood by density gradient centrifugation using Ficoll Paque Plus (GE Healthcare) as described previously [27], [62]. Human PBMC were isolated from venous blood of 3 healthy donors as described previously [27]. Splenocytes were obtained from American Dutch Belted rabbits as previously described [63]. Bovine PBMC were adjusted to a concentration of 1 × 10^6^ cells/ml in complete cell culture medium (RPMI 1640, Gibco) supplemented with 10% heat-inactivated FCS, 100 U/ml penicillin, 100 µg/ml streptomycin, 292 µg/ml L-glutamine (PSG) and 50 µM 2-Mercaptoethanol (Sigma-Aldrich), and cultured at 37°C, 5% CO_2_. Human PBMC (1 × 10^6^) were suspended in RPMI 1640 medium (Life technologies) supplemented with 2% FBS, 100 U/ml penicillin G, and 100 µg/ml streptomycin. The cultures were co-incubated with 10-fold dilutions of rSElX proteins (1 to 10^−6^ µg/ml) for 72 h at 37°C, 5% CO_2_. After adding [^3^H] thymidine (1 µCi), Cells were cultured for a further 18 h and cellular DNA was harvested on glass fiber filters. [^3^H]-thymidine incorporation was quantified by liquid scintillation counting as described previously [27].

### Analysis of Vβ-dependent T-cell activation

Total RNA was extracted from human PBMC prior to and after stimulation with rSElX proteins (1 µg/ml) for 96 h. Expansion of cells expressing different humVβ gene subfamilies was determined using qRT-PCR as described previously [27]. Total RNA was extracted from bovine PBMC before and after stimulation with rSElX variants (1 µg/ml) using Tri-reagent (Sigma–Aldrich). First-strand cDNA was generated from 0.5 µg of RNA using Power SYBR Green RNA-to-CT 2-Step Kit (AB). BovVβ subfamily-specific qRT-PCR primers were designed with Primer3 [64], based on an alignment of bovVβ sequences. cDNA sequences for bovine TRBV genes were derived from the bovine genome and cDNA analyses described by Connelley *et al* [29] (Table S2). To quantify bovVβ subfamily gene expression, qRT-PCR reactions were carried out in 25 µl reactions containing 100ng cDNA, 100 nM primers (Table S2), and SYBR Green I dye master mix (AB) using a Stratagene Mx3000P light cycler. The thermal conditions were 1 cycle at 50°C for 10 min, 1 cycle at 95°C for 10 min, 15 s at 95°C and 1 min at 60°C for 40 cycles. RNA samples were processed in triplicate with NTC and noRT controls. The threshold cycle (C_T_) was determined using MxPro software version 4.1 and normalized to internal controls, β-actin and Constant β (Cβ), by calculating ΔC_T_ [C_T__target_ - C_T β-actin_- C_T (β-actin – Cβ)_]. Normalized ΔC_T_ data were then compared by calculating -ΔΔC_T_ =  -(ΔC_T_ stimulated - ΔC_T_ of unstimulated). Values >0 indicate expansion of particular subset in response to stimulation.

### Statistical analysis

Student's t-test analysis was used to assess differences in fever responses in rabbits and differences in humVβ and bovVβ gene expression. Fisher's exact test was used to assess differences in rabbit survival.

## Supporting Information

Figure S1 ***selx*** **is located in the core genome of** ***S. aureus*** **.** Schematic representation of the genomic context of *selx*. *selx* is depicted in red, the integrase pseudogene is depicted with a blue arrow, white arrows represent hypothetical proteins of unknown function and conserved staphylococcal genes are indicated by black arrows.
(PDF)

Figure S2 **Amino-acid sequence alignment of 16 SElX allelic variants (all alleles except the truncated SElX13).**
(PDF)

Figure S3 **Identification of predicted recombination events among** ***selx*** **alleles.** Coordinates of beginning breakpoints (BB) and end breakpoints (EB) detected by at least 3 different programmes are indicated. Differing filled patterns indicate gene fragments of distinct evolutionary origin. Colored outlines and letters indicate different *selx* alleles.
(PDF)

Figure S4 **Phylogenetic tree of** ***selx*** **.** A neighbour joining tree based on *selx* gene sequences has a distinct topology to a concatenated multilocus sequence-based tree (Fig. 1b). Bootstrap values greater than 40 are indicated.
(PDF)

Figure S5 **Western blot analysis indicates a lack of cross-reactivity of SElX antibodies for TSST-1 or SSL7.** Western blot analysis of recombinant SElX TSST-1, SSL7 with SElX-specific antisera raised in rats and serum samples from human and ovine infections.
(PDF)

Figure S6 **Phenotypic analysis of the LAC Δ** ***selx*** **mutant and LAC Δ** ***selx*** **rep.** a) Western blot analysis of LAC wt, LAC Δ*selx*, and LAC Δ*selx* repaired, with SElX-specific antibody. b) Hemolytic titration of LAC wt, LAC Δ*selx* mutant and LAC Δ*selx* repaired supernatants incubated with washed rabbit erythrocytes. Hemolytic titre was determined to be the reciprocal of the dilution which resulted in ∼50% hemolysis (circled in black). c) SDS PAGE analysis of concentrated supernatant protein fractions resulted in indistinguishable profiles. In addition, quantification of α-toxin and PVL levels in LAC wt and LAC Δ*selx* supernatants revealed identical toxin levels at 3 h, 6 h, 8 h, and 24 h time-points during growth in CCY medium by specific enzyme-linked immunosorbent assays (data not shown) (α-toxin, bioMerieux, Nabi Biopharmaceuticals, and PVL; Besseyre des Horts et al, Infect Immun. 2010 78:260-4).
(PDF)

Table S1 **Distribution and coordinates of** ***selx*** **in sequenced** ***S. aureus*** **genomes.**
(DOC)

Table S2 **Oligonucleotide primers used in this study.**
(DOC)

Table S3 ***S. aureus*** **strains employed and** ***selx*** **genotype and phenotype.**
(DOC)
