# Supplementary figures and images for: A Novel Core Genome-Encoded Superantigen Contributes to Lethality of Community-Associated MRSA Necrotizing Pneumonia
Source: PLoS Pathog. 2011 Oct 13;7(10):e1002271. doi: 10.1371/journal.ppat.1002271 (PMC3192841; doi:10.1371/journal.ppat.1002271)

Fig.S1

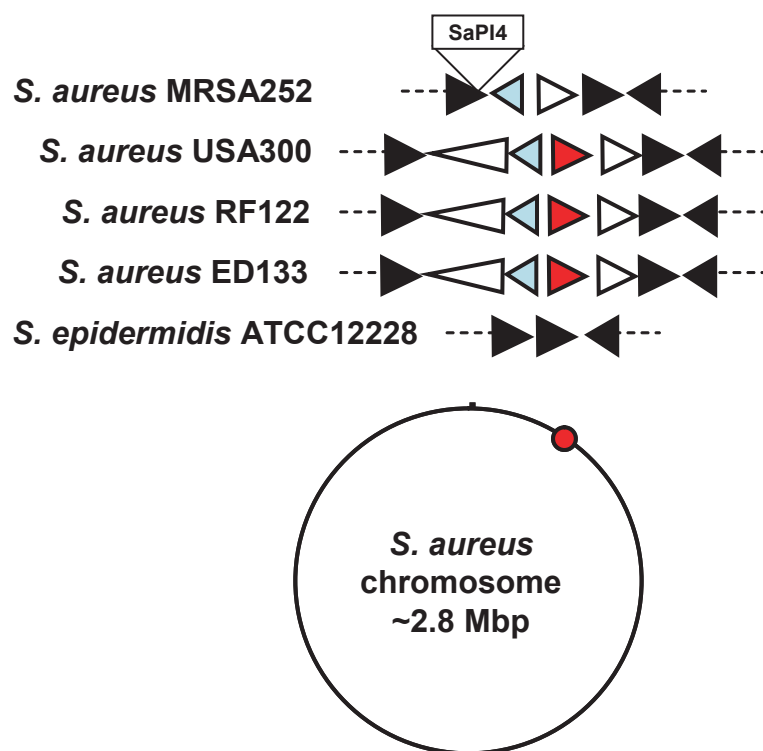

Supplement: Figure S1 — selx is located in the core genome of S. aureus . Schematic representation of the genomic context of selx. selx is depicted in red, the integrase pseudogene is depicted with a blue arrow, white arrows represent hypothetical proteins of unknown function and conserved staphylococcal genes are indicated by black arrows. (PDF) [file ppat.1002271.s001.pdf]

Fig S2

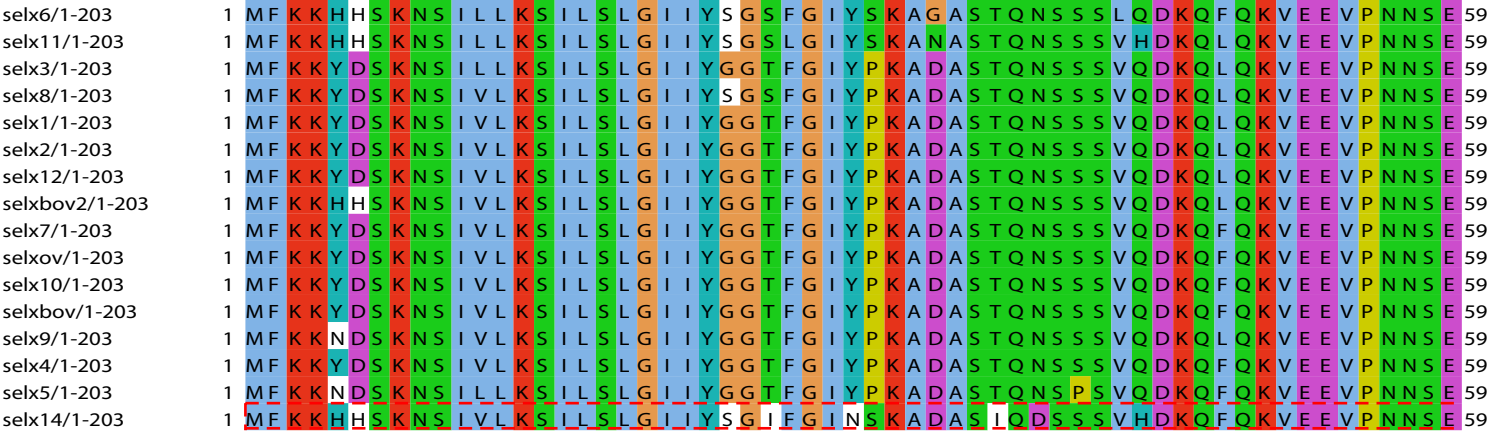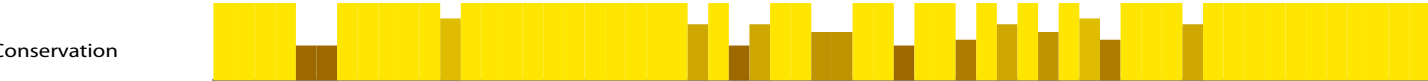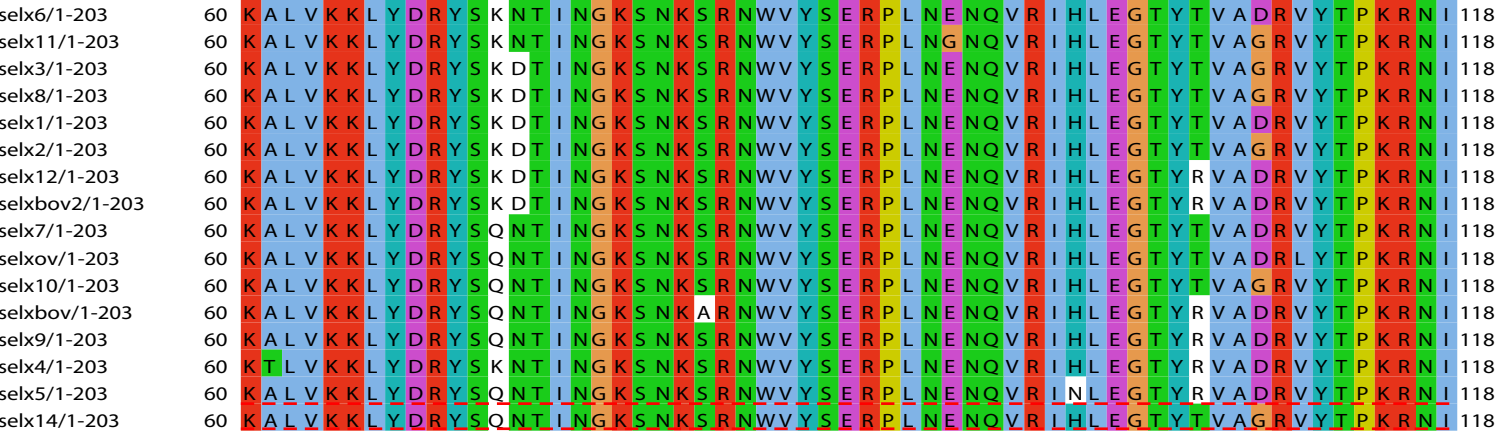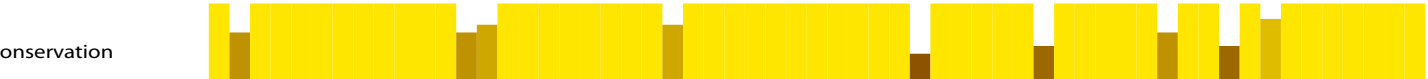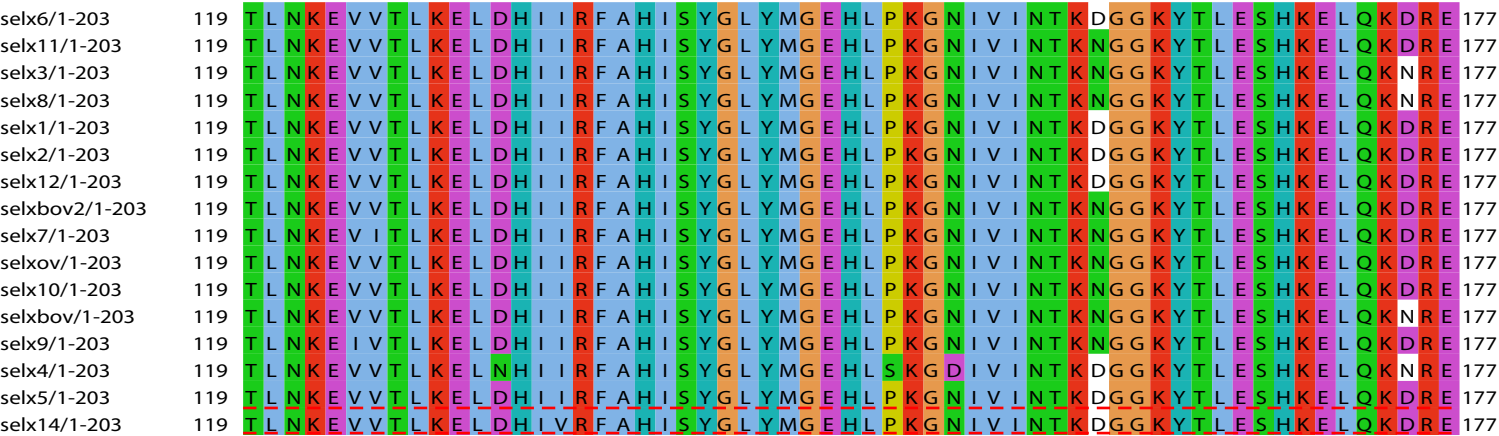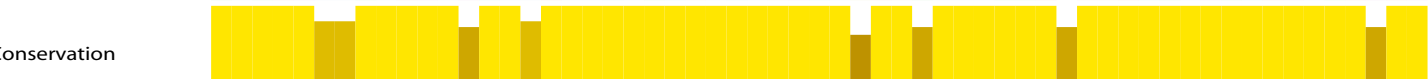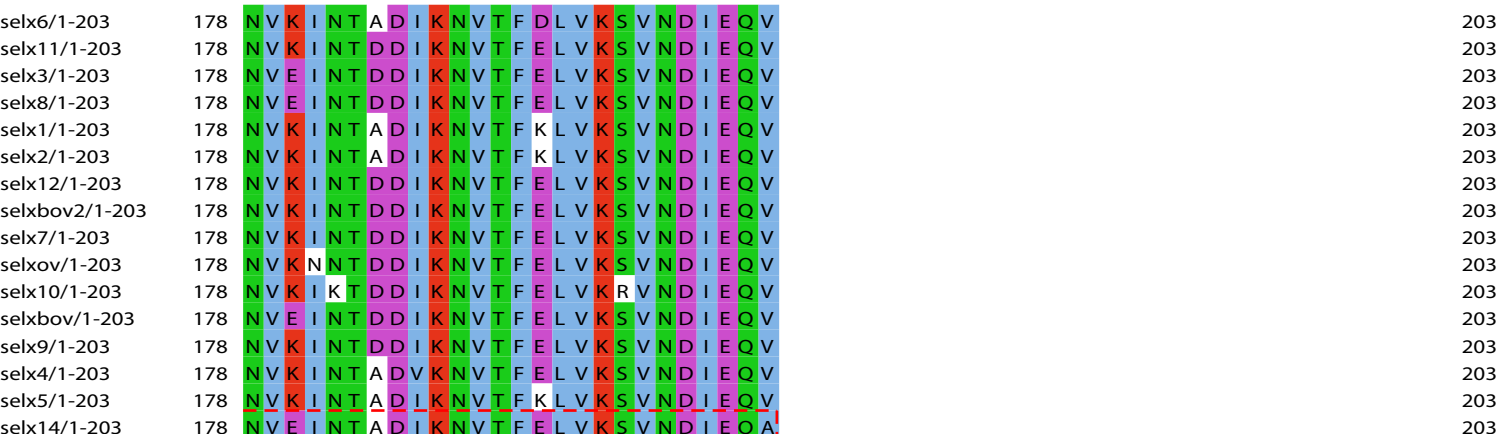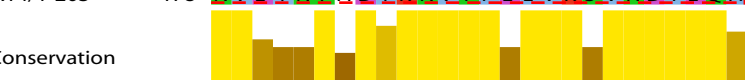

Supplement: Figure S2 — Amino-acid sequence alignment of 16 SElX allelic variants (all alleles except the truncated SElX13). (PDF) [file ppat.1002271.s002.pdf]

Fig. S3

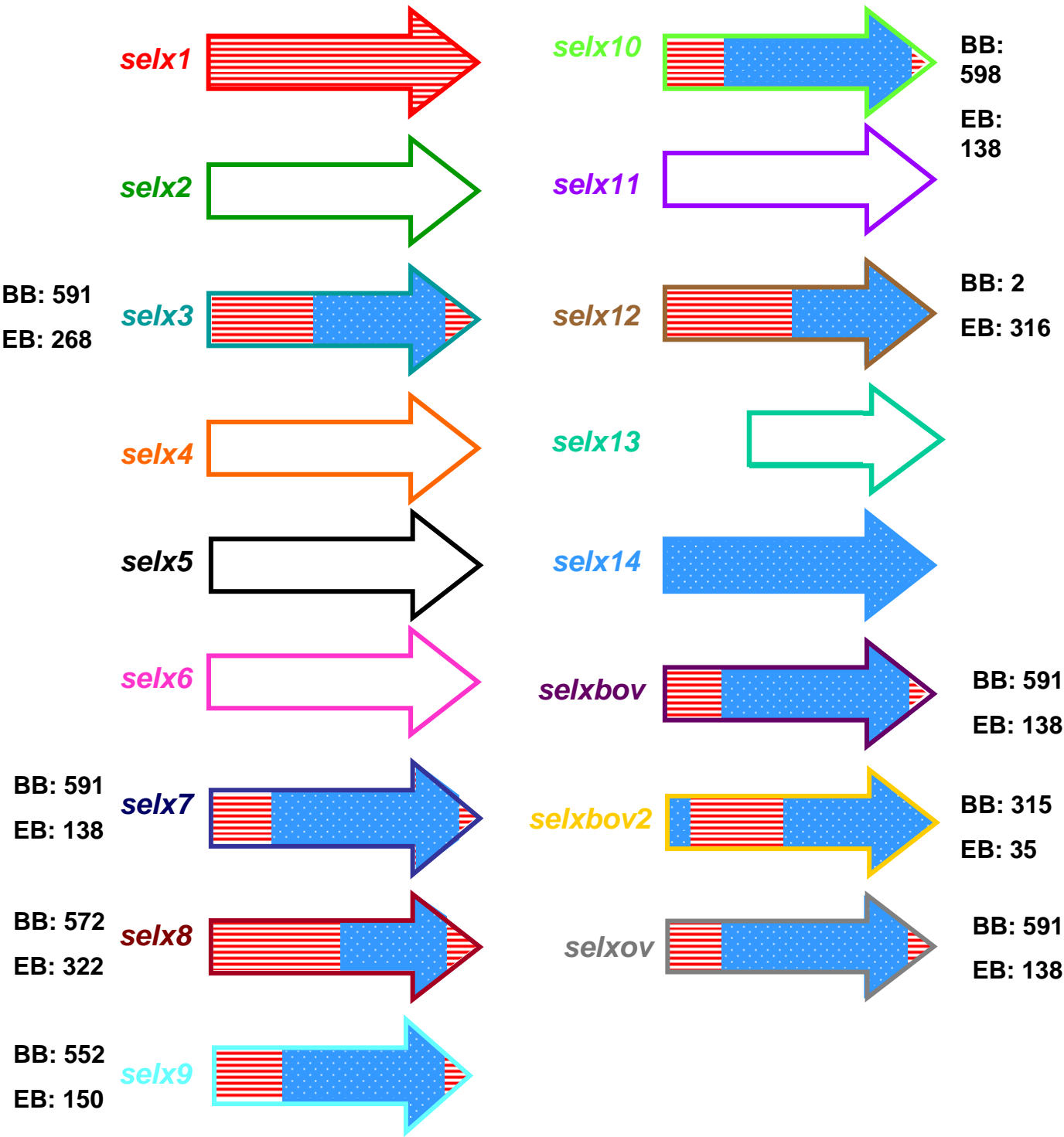

Supplement: Figure S3 — Identification of predicted recombination events among selx alleles. Coordinates of beginning breakpoints (BB) and end breakpoints (EB) detected by at least 3 different programmes are indicated. Differing filled patterns indicate gene fragments of distinct evolutionary origin. Colored outlines and letters indicate different selx alleles. (PDF) [file ppat.1002271.s003.pdf]

Fig S4

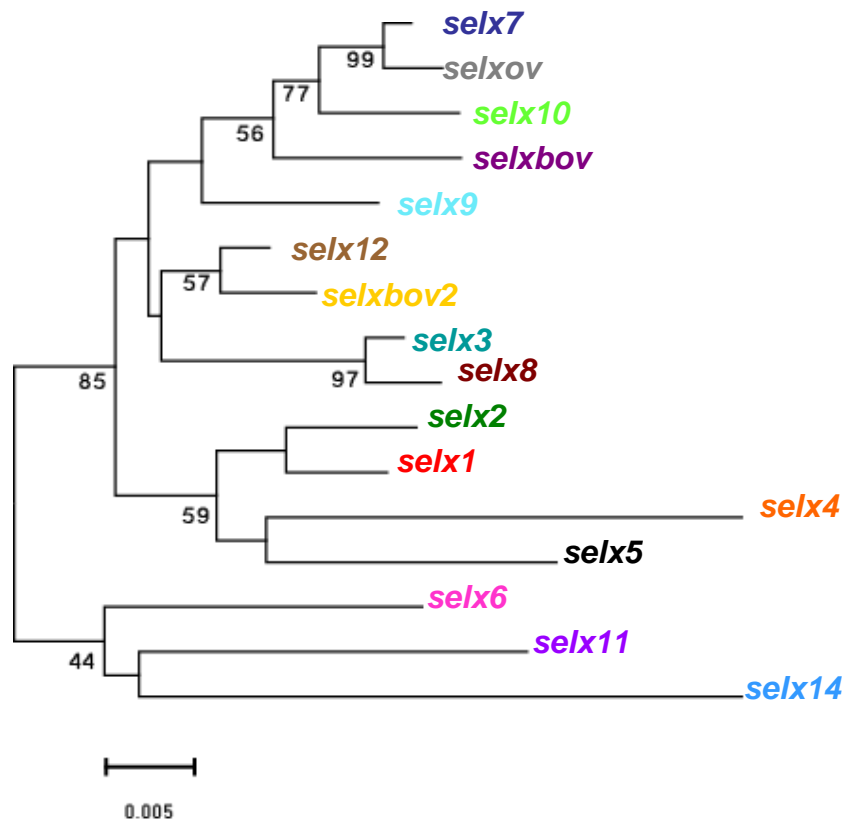

Supplement: Figure S4 — Phylogenetic tree of selx . A neighbour joining tree based on selx gene sequences has a distinct topology to a concatenated multilocus sequence-based tree (Fig. 1b). Bootstrap values greater than 40 are indicated. (PDF) [file ppat.1002271.s004.pdf]

Fig. S5

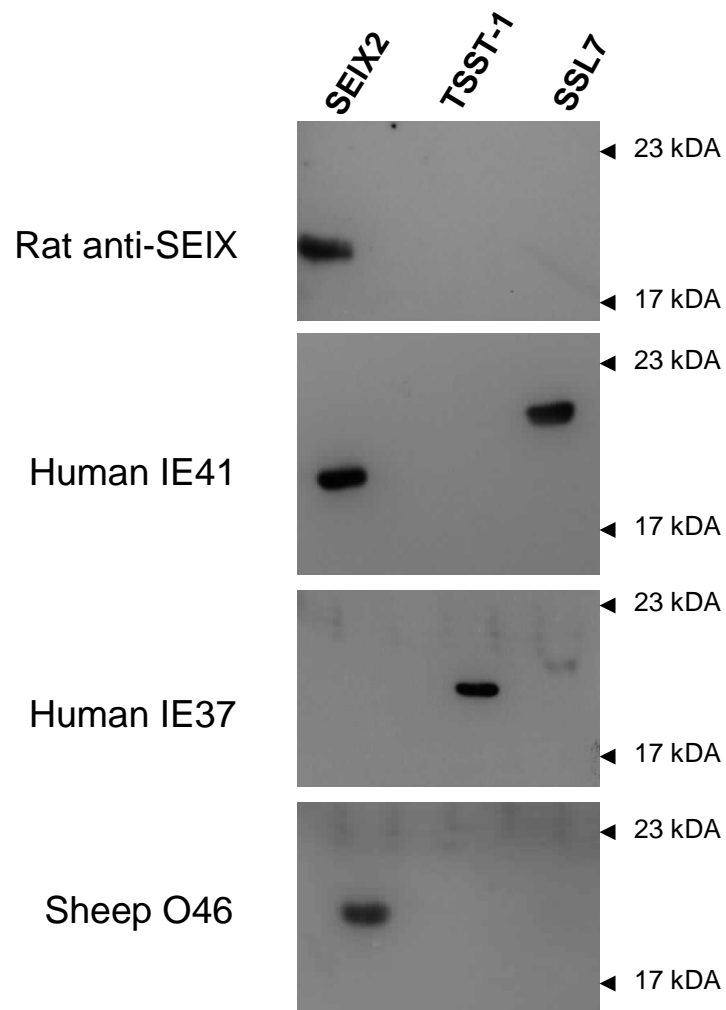

Supplement: Figure S5 — Western blot analysis indicates a lack of cross-reactivity of SElX antibodies for TSST-1 or SSL7. Western blot analysis of recombinant SElX TSST-1, SSL7 with SElX-specific antisera raised in rats and serum samples from human and ovine infections. (PDF) [file ppat.1002271.s005.pdf]

Fig. S6

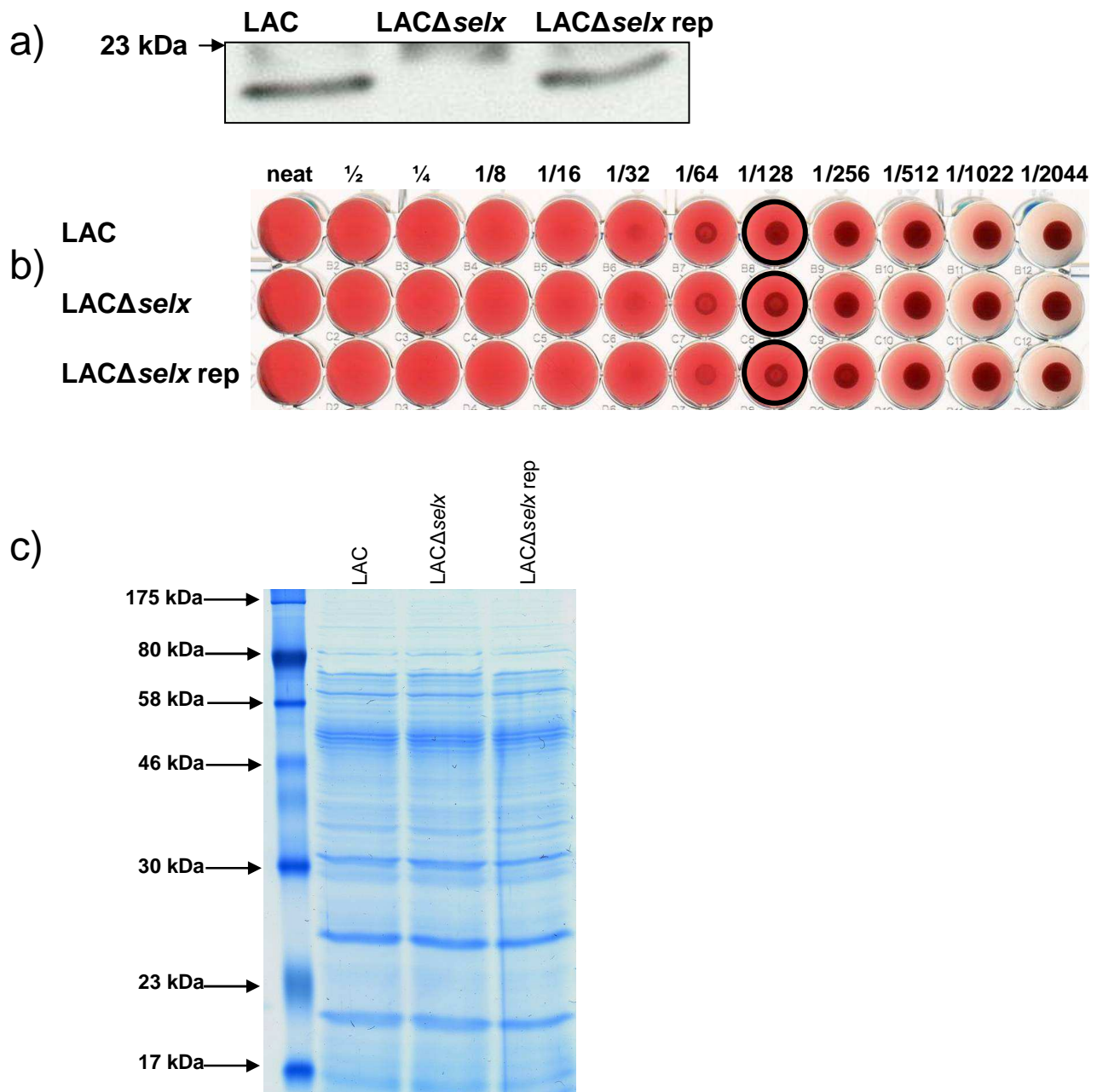

Supplement: Figure S6 — Phenotypic analysis of the LAC Δ selx mutant and LAC Δ selx rep. a) Western blot analysis of LAC wt, LAC Δselx, and LAC Δselx repaired, with SElX-specific antibody. b) Hemolytic titration of LAC wt, LAC Δselx mutant and LAC Δselx repaired supernatants incubated with washed rabbit erythrocytes. Hemolytic titre was determined to be the reciprocal of the dilution which resulted in ∼50% hemolysis (circled in black). c) SDS PAGE analysis of concentrated supernatant protein fractions resulted in indistinguishable profiles. In addition, quantification of α-toxin and PVL levels in LAC wt and LAC Δselx supernatants revealed identical toxin levels at 3 h, 6 h, 8 h, and 24 h time-points during growth in CCY medium by specific enzyme-linked immunosorbent assays (data not shown) (α-toxin, bioMerieux, Nabi Biopharmaceuticals, and PVL; Besseyre des Horts et al, Infect Immun. 2010 78:260-4). (PDF) [file ppat.1002271.s006.pdf]
