# Supplementary material for: A Novel Core Genome-Encoded Superantigen Contributes to Lethality of Community-Associated MRSA Necrotizing Pneumonia
Source: PLoS Pathog. 2011 Oct 13;7(10):e1002271. doi: 10.1371/journal.ppat.1002271 (PMC3192841; doi:10.1371/journal.ppat.1002271)
Supplement: Table S1 — Distribution and coordinates of selx in sequenced S. aureus genomes. (DOC) [file ppat.1002271.s007.doc]

**Table S1:** Distribution and coordinates of *selx* in sequenced *S. aureus* genomes.

| **Strain** | **Reference number** | **ST** | ***selx* allele** | **Locus tag/ coordinates ^a, b^** |
| --- | --- | --- | --- | --- |
| MSSA476 | NC_002953.3 | 1 | 5 | SAS0347 |
| MW2 | NC_003923.1 | 1 | 5 | MW0345 |
| TCH70 | ACHH01000007.1 | 1 | 5 | 12962-12351 |
| 51811 | ADVP01000041 | 1 | 5 | 45075-45686 |
| N315 | NC_002745.2 | 5 | 1 | SA0357 |
| Mu50 | NC_002758.2 | 5 | 1 | SAV0370 |
| Mu50_omega | BABM01000001.1 | 5 | 1 | 415121-415732 |
| Mu3 | NC_009782 | 5 | 1 | SAHV_0367 |
| CF marsielle | CABA01000104.1 | 5 | 1 | 34300-33689 |
| MR1 | ACZQ01000042.1 | 5 | 1 | 21763-21152 |
| ED98 | NC_013450.1 | 5 | 1 | 377804-378415 |
| A10102 | ACSO01000006.1 | 5 | 1 | 79351-79962 |
| A5937 | ACKC01000031.1 | 5 | 1 | 354030-354641 |
| A6224 | ACKE01000012.1 | 5 | 1 | 48160-48771 |
| A9781 | ACKL01000029.1 | 5 | 1 | 9191-9802 |
| A6300 | ACKF01000002.1 | 5 | 1 | 53512-54123 |
| A9299 | ACKH01000031.1 | 5 | 1 | 89178-88567 |
| A9719 | ACKJ01000043 | 5 | 1 | 261259-261870 |
| A8155 | ACKG01000031.1 | 5 | 1 | 261394-262005 |
| A9763 | ACKK01000029.1 | 5 | 1 | 89367-88756 |
| A5948 | ACKD01000059.1 | 8 | 2 | 35867-35256 |
| A9754 | ADJI01000010 | 8 | 2 | 70578-71175 |
| A9765 | ACSN01000037 | 8 | 2 | 4665-5262 |
| TCHFPR3757 | NC_007793.1 | 8 | 2 | SAUSA300_0370 |
| TCH1516 | NC_010079.1 | 8 | 2 | USA300HOU_0392 |
| Newman | NC_009641.1 | 8 | 2 | NWMN_0362 |
| NCTC8325 | NC_007795.1 | 8 | 2 | SAOUHSC_00354 |
| 132 | ACOT01000013.1 | 8 | 2 | 85792-86403 |
| D30 | ABFB01000008.1 | 8 | 2 | 27371-26760 |
| 930918-3 | ABFA01000030.1 | 8 | 2 | 34590-33979 |
| H19 | ACSS01000018.1 | 10 | 7 | 252814-253425 |
| D139 | ACSR01000010.1 | 10 | 7 | 3446-4057 |
| NOH4 | Sanger | 22 slv | 6 | NK |
| EMRSA-15 | Sanger | 22 | 6 | NK |
| TCH60 | ACHC01000008.1 | 30 | Not present | N/A |
| MRSA252 | NC_002952.2 | 36 | Not present | N/A |
| M876 | ACJV01000012.1 | 30 | Not present | N/A |
| MN8 | ACJA01000076.1 | 30 | Not present | N/A |
| A9635 | ACKI01000032.1 | 45slv | 4 | 90134-90562 |
| TCH130 | ACHD01000271.1 | 72 | 5 | 27060-27671 |
| JKD6159 | CP002114 | 93 | 12 | 396778-397389 |
| JH1 | NC_009632.1 | 105 | 1 | SaurJH1_0429 |
| JH9 | NC_009487.1 | 105 | 1 | SaurJH9_0419 |
| ED133 | CP001996 | 133 | ov | 415169-415780 |
| RF122 | NC_007622 | 151 | bov1 | SAB0321 |
| 04-02891 | CP001844 | 225 | 1 | 414287-414898 |
| **Strain** | **Reference number** | **ST** | ***Selx* allele** | **Locus tag/ coordinates ^a, b^** |
| 0582/ TW20 | Sanger | 239 | 2 | N/A |
| JKD6008 | ABRZ01000012.1 | 239 | 2 | SAA6008_00370 |
| JKD6009 | ABSA01000060.1 | 239 | 2 | 5327-5938 |
| BAA-39 | AEEK01000046 | 239 | 2 | 259791-260388 |
| COL | NC_002951.2 | 250 | 2 | SACOL0442 |
| S0385 | AM990992 | 398 | 13 | 464029- 464344 |
| TCH959 | AASB02000040.1 | 1159 | 3 | 71002 - 71613 |

**^a^** N/A, not applicable.

**^b^** NK, not known
