# Supplementary material for: A Novel Core Genome-Encoded Superantigen Contributes to Lethality of Community-Associated MRSA Necrotizing Pneumonia
Source: PLoS Pathog. 2011 Oct 13;7(10):e1002271. doi: 10.1371/journal.ppat.1002271 (PMC3192841; doi:10.1371/journal.ppat.1002271)
Supplement: Table S2 — Oligonucleotide primers used in this study. (DOC) [file ppat.1002271.s008.doc]

**Table S2.** Oligonucleotide primers used in this study

| **Primer** | | **Forward Sequence (5′-3′) ^a^** | **Reverse Sequence (3′-5′) ^a^** |  |
| --- | --- | --- | --- | --- |
| *selx* | | AGCAGACGCGTCAACACAAA | ACTTGTTCAATGTCATTAACACTTTTCAC |  |
| *selx*seq | | TGGTAGCAAATTAAAGTTAATCAAGAG | TGCTAATCATAACAAAGAAAGCTAGG |  |
| *selx*q | | AGCAGACGCGTCAACACACAAA | GGTCTCTCTGAATAAACCCAATTCC |  |
| *16SrRNA* | | TATGGAGGAACACCAGTGGCGAAG | TCATCGTTTACGGCGTGGACTACC |  |
| *selx*pET | | TAGCCATATGTCAACACAAAATTCCTCAA | GCGCGGATCCTCAAACTTGTTCAATGTC |  |
| selx AB | | AGTCGATGCTTTGGGTAGTGAG | TAATTACCTCCTTGATGTAAAGC |  |
| selx CD | | GCTTTACATCAAGGAGGTAATTATATCGCTAATACTTTGAAAGTTAGG | tcaaatgtagcagtatacattaattgcg |  |
| rep AB | | TGTCTCCTTTACTCCGAATGCTC | TCTGCCAGCGACTGTGTATG |  |
| rep CD | | CATACACAGTCGCTGGCAGAGTGTATACACCTAAGAGG | TCATCCATTCTAGTAGACACCTCTGTC |  |
| EZ | | ATGTGGCTAATTTTGTTCGAGTCG | TTAATCGTCATGCGTTACTTTCGTTCG |  |
| Vβ 1 | | ctgatcaaatcaagaaaascagcaag | ctgagtctgycagctccaag |  |
| Vβ 2 | | cgctctcgtctctcagcag | taaccttgttcgtatgtggcatc |  |
| Vβ 3 | | gctgctccatttctcaatcga | ggcacatccccttcctcaa |  |
| Vβ 4 | | ctcctgggactgggttctg | agccctgattggcagtagc |  |
| Vβ 5 | | cctatgtctggacacagcagtg | tcgaaaagtttcctttagctctttg |  |
| Vβ 6 | | gggaggggccagactgtaa | tttaggcatycctgatttgtc |  |
| Vβ 7 | | ctcagataccaaaatacctagtcatg | gaacactctcatttccagtgagtttc |  |
| Vβ 8 | | gtcactctgagttgtgagcyga | ccgactcatcaataggagcttg |  |
| Vβ 9 | | cctcacacagatgggaactaagac | gcaccgtttcatttccaacg |  |
| Vβ 10 | | agcaaagatggattgtgtccc | ggtggagttgatttccaggct |  |
| Vβ 11 | | tcaaactatgggccttgacaac | gctctcctttctccgtggtattaac |  |
| Vβ 13 | | ccakgtcgtgaggasagga | ctgagtaatggatyagcctcag |  |
| Vβ 15 | cacaggaaagagcactgtactgg | | catctcctttgttaatatcatcgatacc | |
| Vβ 16 | | gaccctatttctggacatgaatct | cctttgggcatccctgagt |  |
| Vβ 17 | | gtgaccctggartgtraacag | tcwcgagagrcrctgtagc |  |
| Vβ 24 | | taccgggctgggaaaacc | tgtttttttccttggtgatttgttta |  |
| Vβ 28 | | cgcaggatcttggagactga | tcttaaaggagtcagggccagtt |  |
| Vβ X | | ctacaggtgctggccagtctg | ggtttatgacttccttatccccgg |  |
| Constant β | | cctggtgggtgaacaggaag | cctcccactggtcctggtc |  |
| β-actin | | ggccgagcggaaatcg | gccatctcctgctcgaagtc |  |

**^a^** Restriction sites incorporated are underlined.
