# Supplementary material for: A Novel Core Genome-Encoded Superantigen Contributes to Lethality of Community-Associated MRSA Necrotizing Pneumonia
Source: PLoS Pathog. 2011 Oct 13;7(10):e1002271. doi: 10.1371/journal.ppat.1002271 (PMC3192841; doi:10.1371/journal.ppat.1002271)
Supplement: Table S3 — S. aureus strains employed and selx genotype and phenotype. (DOC) [file ppat.1002271.s009.doc]

| **Strain**  **Table S3** *S. aureus* strains employed, and *selx* genotype and phenotype | **Host/**  **Characteristic** | **Geographic origin, year** | **MLST/ PFGE/MLEE Type** | **Reference/**  **Source** | **Presence of *selx/* allele*** | **SElX expression** |
| --- | --- | --- | --- | --- | --- | --- |
| RF103 | Bovine | Ireland | 71 | [1] | + | ND |
| RF31 | Bovine | Ireland | 97 | Fitzgerald J.R | +/ bov2 | ND |
| CTH108 | Bovine | USA | 115 | [2] | + | ND |
| MSA948 | Bovine | USA | 126 | Musser J.M | + | ND |
| 951 | Bovine | USA | 126 | [3] | +/ 8 | ND |
| DS36 (V329) | Bovine | Sweden | 133 | [4] | + | ND |
| RF287 | Bovine | Ireland, 1986 | 133 | Fitzgerald J.R | + | + |
| VI50901 | Bovine | Norway­­­ | 133 | [5] | + | + |
| C123/5/005 | Bovine | UK, 2003 | 151 | [6] | + | ND |
| RF122 | Bovine | Ireland, 1993 | 151 | [1] | +/ bov1 | + |
| 38963 | Bovine | UK | 151 | [6] | + | ND |
| RF113 | Bovine | Ireland | 151 | Fitzgerald J.R | + | + |
| MSA1011 | Bovine | USA | 352 | Musser J.M | +/ bov2 | ND |
| MSA961 | Bovine | USA | ET146 | [7] | + | ND |
| T2 (2521) | Bovine | Ireland | ND | Smyth C. | + | ND |
| T6 (2242) | Bovine | Ireland | ND | Smyth C. | + | ND |
| T7 (2242) | Bovine | Ireland | ND | Smyth C. | + | ND |
| T12 (2242) | Bovine | Ireland | ND | Smyth C. | + | ND |
| T21 (2487) | Bovine | Ireland | ND | Smyth C. | + | ND |
| T22 (2487) | Bovine | Ireland | ND | Smyth C. | + | ND |
| ED98 | Chicken | N. Ireland | 5 | [15] | +/ 2 | - |
| Newman | Human | UK | 8 | [8] | + | + |
| 434 (C434) | Human | UK,1997 | 8 | [9] | + | ND |
| 126 (C126P) | Human | UK, 1997 | 12 | [9] | +/ 2 | ND |
| 3150 | Human | UK | 15 | [10] | + | ND |
| 3158 | Human | UK | 15 | [10] | +/ 2 | ND |
| 3060 | Human | UK | 15 | [10] | + | ND |
| C207 | Human | UK, 1997 | 15 | [11] | +/ 2 | ND |
| H383 | Human | UK, 1997 | 22 | [9] | + | ND |
| 03-2372.2 #4 | Human MRSA | UK | 22 | Scottish MRSA Ref. Lab. (SMRSARL) | + | ND |
| 07-8898.11 #10 | Human MRSA | UK | 22 | SMRSARL | + | ND |
| **Strain** | **Host/**  **Characteristic** | **Geographic origin, year** | **MLST/ PFGE/MLEE Type** | **Reference/**  **Source** | **Presence of *selx/* allele*** | **SElX expression** |
| MSA1832 | Human | USA, 1968 | 30 | [12] | - | ND |
| 02-2008.L #23 | Human MRSA | UK | 36 | SMRSARL | - | ND |
| MSA2389 | Human furunculosis | Sweden | 45 (ET39) | [12] | +/ 4 | ND |
| MSA2020 | Human SSS | France | 121 | [12] | +/ 10 | + |
| MSA1601 | Human MRSA | USA, 1980s | ET53 | [7] | + | ND |
| MSA3418 | Human MRSA | Australia, 1980s | ET89 | [7] | + | ND |
| MSA3400 | Human MRSA | Ireland, 1990 | ET91 | [7] | + | ND |
| MSA890 | Human | USA MRSA | ET93 | [7] | + | ND |
| MSA820 | Human MRSA | USA | ET93 | [7] | + | ND |
| MSA3410 | Human MRSA | UK, 1960s | ET93 | [7] | + | ND |
| MSA1695 | Human SSS | Japan | ET93 | [12] | + | ND |
| MSA2120 | Human | Denmark, 1983, | ET146 | [12] | + | ND |
| MSA2965 | Human Sepsis | Canada, 1983 | ET191 | [12] | + | + |
| MSA537 | Human | USA, 1985,TSS | ET234 | [12] | + | ND |
| LAC | Human CA-MRSA | USA | USA300 | [13] | + | + |
| LACΔ*selx* | *selx-*deficient mutant | USA | USA300 | This study | - | - |
| LACΔ*selx*rep | Selx-repaired | USA | USA300 | This study | + | + |
| 88006 | Human CA-MRSA | USA | USA300 | [14] | + | ND |
| 88007 | Human CA-MRSA | USA | USA300 | [14] | + | ND |
| 88008 | Human CA-MRSA | USA | USA300 | [14] | + | + |
| 88009 | Human CA-MRSA | USA | USA300 | [14] | + | ND |
| 88010 | Human CA-MRSA | USA | USA300 | [14] | + | + |
| DS30 (St153) | Caprine | Italy | 22 | [15] | + | ND |
| DS27 (St125) | Caprine | Italy | 25 | [15] | +/ 9 | ND |
| **Strain** | **Host** | **Geographic origin, year** | **MLST/ PFGE/MLEE Type** | **Reference/**  **Source** | **Presence of *selx/* allele*** | **SElX expression** |
| VI50895 | Caprine | Norway | 130 | [5] | + | + |
| DS74 | Caprine | Norway | 133 | [16] | + | ND |
| DS28 | Caprine | Italy | 133 | [15] | + | ND |
| VI50896 | Caprine | Norway | 481 | [5] | + | ND |
| ED133 | Ovine | France, 97 | 133 | [17] | + | + |
| DS13 (St7) | Ovine | Italy | 133 | [15] | + | ND |
| DS83 (891-1) | Ovine | Norway | 133 | [16] | + | + |
| DS102(6659-2) | Ovine | Sweden | 151 slv | [16] | + | ND |
| DS95 (1) | Ovine | Denmark | 706 (9slv) | [16] | + | + |
| VET-BZ30 | Ovine | Brazil, 2003 | 750 | [18] | + | - |
| MSA535 | Ovine mastitis | Germany | F1\ ET66 | [12] | + | ND |
| B40 | Swine | Hong Kong | 9 | [19] | +/ 11 | ND |

* Allele numbers for selected representative strains are included

^Ψ^ +, positive; -, negative; ND, not done

**References:**

1. Fitzgerald JR, Meaney WJ, Hartigan PJ, Smyth CJ, Kapur V (1997) Fine-structure molecular epidemiological analysis of Staphylococcus aureus recovered from cows. Epidemiol Infect 119: 261-269.

2. Smith EM, Green LE, Medley GF, Bird HE, Fox LK, et al. (2005) Multilocus sequence typing of intercontinental bovine Staphylococcus aureus isolates. J Clin Microbiol 43: 4737-4743.

3. Sischo WM, Heider LE, Miller GY, Moore DA (1993) Prevalence of contagious pathogens of bovine mastitis and use of mastitis control practices. J Am Vet Med Assoc 202: 595-600.

4. Cucarella C, Solano C, Valle J, Amorena B, Lasa I, et al. (2001) Bap, a Staphylococcus aureus surface protein involved in biofilm formation. J Bacteriol 183: 2888-2896.

5. Jorgensen HJ, Mork T, Caugant DA, Kearns A, Rorvik LM (2005) Genetic variation among Staphylococcus aureus strains from Norwegian bulk milk. Appl Environ Microbiol 71: 8352-8361.

6. Sung JM, Lloyd DH, Lindsay JA (2008) Staphylococcus aureus host specificity: comparative genomics of human versus animal isolates by multi-strain microarray. Microbiology 154: 1949-1959.

7. Musser JM, Kapur V (1992) Clonal analysis of methicillin-resistant Staphylococcus aureus strains from intercontinental sources: association of the mec gene with divergent phylogenetic lineages implies dissemination by horizontal transfer and recombination. J Clin Microbiol 30: 2058-2063.

8. Duthie ES (1952) Variation in the antigenic composition of staphylococcal coagulase. J Gen Microbiol 7: 320-326.

9. Enright MC, Day NP, Davies CE, Peacock SJ, Spratt BG (2000) Multilocus sequence typing for characterization of methicillin-resistant and methicillin-susceptible clones of Staphylococcus aureus. J Clin Microbiol 38: 1008-1015.

10. Lindsay JA, Moore CE, Day NP, Peacock SJ, Witney AA, et al. (2006) Microarrays reveal that each of the ten dominant lineages of Staphylococcus aureus has a unique combination of surface-associated and regulatory genes. J Bacteriol 188: 669-676.

11. Feil EJ, Cooper JE, Grundmann H, Robinson DA, Enright MC, et al. (2003) How clonal is Staphylococcus aureus? J Bacteriol 185: 3307-3316.

12. Musser JM, Selander RK (1990); Novick RP, editor. New York VCH pp 59-67

13. Voyich JM, Otto M, Mathema B, Braughton KR, Whitney AR, et al. (2006) Is Panton-Valentine leukocidin the major virulence determinant in community-associated methicillin-resistant Staphylococcus aureus disease? J Infect Dis 194: 1761-1770.

14. Kennedy AD, Otto M, Braughton KR, Whitney AR, Chen L, et al. (2008) Epidemic community-associated methicillin-resistant Staphylococcus aureus: recent clonal expansion and diversification. Proc Natl Acad Sci U S A 105: 1327-1332.

15. Foschino R, Invernizzi A, Barucco R, Stradiotto K (2002) Microbial composition, including the incidence of pathogens, of goat milk from the bergamo region of italy during a lactation year. J Dairy Res 69: 213-225.

16. Mork T, Tollersrud T, Kvitle B, Jorgensen HJ, Waage S (2005) Comparison of Staphylococcus aureus genotypes recovered from cases of bovine, ovine, and caprine mastitis. J Clin Microbiol 43: 3979-3984.

17. Ben Zakour NL, Guinane CM, Fitzgerald JR (2008) Pathogenomics of the staphylococci: insights into niche adaptation and the emergence of new virulent strains. FEMS Microbiol Lett 289: 1-12.

18. Aires-de-Sousa M, Parente CE, Vieira-da-Motta O, Bonna IC, Silva DA, et al. (2007) Characterization of Staphylococcus aureus isolates from buffalo, bovine, ovine, and caprine milk samples collected in Rio de Janeiro State, Brazil. Appl Environ Microbiol 73: 3845-3849.

19. Guardabassi L, O'Donoghue M, Moodley A, Ho J, Boost M (2009) Novel lineage of methicillin-resistant Staphylococcus aureus, Hong Kong. Emerg Infect Dis 15: 1998-2000.
